# Antisecretory Factor 16 (AF16): A Promising Avenue for the Treatment of Traumatic Brain Injury—An In Vitro Model Approach

**DOI:** 10.1007/s12031-024-02268-6

**Published:** 2024-11-07

**Authors:** Nicola Vahrmeijer, Jurgen Kriel, Bradley M. Harrington, Anton Du Preez van Staden, Adriaan Johannes Vlok, Lize Engelbrecht, Andre Du Toit, Ben Loos

**Affiliations:** 1 Department of Physiological Sciences, Stellenbosch University, Merriman Avenue, Mike de Vries Building, Stellenbosch, 7600 South Africa; 2 Central Analytical Facilities, Stellenbosch University, Tygerberg Medical Campus, Clinical Building, 7Th Floor, Room 7063, Stellenbosch, South Africa; 3 Department of Neurosurgery, Tygerberg University Hospital, Tygerberg, Cape Town South Africa; 4 Division Clinical Pharmacology, Department of Medicine, Faculty of Medicine and Health Sciences, Stellenbosch University, Cape Town, South Africa; 5 Central Analytical Facilities, Stellenbosch University, Merriman Avenue, Mike de Vries Building, Stellenbosch, 7600 South Africa

**Keywords:** Traumatic brain injury (TBI), Antisecretory factor (AF16), Autophagy, Mitochondrial dynamics, Correlative light and electron microscopy (CLEM)

## Abstract

Traumatic brain injury (TBI) is caused by an external mechanical force to the head, resulting in abnormal brain functioning and clinical manifestations. Antisecretory factor (AF16) is a potential therapeutic agent for TBI treatment due to its ability to inhibit fluid secretion and decrease inflammation, intracranial pressure, and interstitial fluid build-up, key hallmarks presented in TBI. Here, we investigated the effect of AF16 in an in vitro model of neuronal injury, as well as its impact on key components of the autophagy pathway and mitochondrial dynamics. N2A^wt^ cells were treated with AF16, injured using a scratch assay, and analysed using confocal microscopy, correlative light and electron microscopy (CLEM), flow cytometry, and western blotting. Our results reveal that AF16 enhances autophagy activity, regulates mitochondrial dynamics, and provides protection as early as 6 h post-injury. Fluorescently labelled AF16 was observed to localise to lysosomes and the autophagy compartment, suggesting a role for autophagy and mitochondrial quality control in conferring AF16-associated neuronal protection. This study concludes that AF16 has potential as a therapeutic agent for TBI treatment through is regulation of autophagy and mitochondrial dynamics.

**Supplementary Information:**

The online version contains supplementary material available at 10.1007/s12031-024-02268-6.

## Introduction

Traumatic brain injury (TBI) is a major world-wide health burden, commonly referred to as the silent epidemic (Dewan et al. 2019; Salehi et al. 2017). TBI results from an external mechanical impact to the head which causes acquired brain dysfunction (Kumaria 2017) together with subsequent clinical manifestations such as headaches, nausea, vomiting, and seizures (Morrison et al. 1998). Despite the incomprehensive data available, it is estimated that in developed countries TBI annually affects nearly 69 million people globally resulting in either permanent disability or death (Dewan et al. 2019). TBI in developing countries on the other hand, such as South Africa, is estimated to account for 150–170 cases per 100,000 compared to the global average of 100 cases per 100,000 (Buitendag et al. 2018). The leading cause of TBI differs with regards to geographical location with the majority of global TBI cases attributed to motor vehicle accidents, whilst in developing countries, including South Africa, gunshot wounds and assault injuries are more notable causes of TBI (Hyder et al. 2007; Coronado et al. 2012; Asemota et al. 2013; Khan et al. 2015; Iaccarino et al., 2018).

TBI is typically categorised into two injury phases, a primary mechanical injury and a downstream secondary injury comprised of a complex cellular and molecular pathophysiology (Kumaria 2017; Sulhan et al. 2020; Zeng et al. 2020). Each phase is defined by its own distinct downstream responses. The primary insult associated with TBI results from the physical and mechanical impact to the brain and is characterised by direct neuronal cell loss and necrotic cell death followed by the disruption of the blood brain barrier (BBB) and development of oedema which in turn disrupts brain homeostasis (Puntambekar et al. 2018). The secondary post-insult cell death, oedema, and resulting increased intracranial pressure (ICP), promote secondary injury cascades, which drive various cell death subroutines. These cascades can be initiated anytime from hours to days following the initial insult (McKee & Daneshvar, 2015). The secondary injury is heterogenous in nature, due to its multifaceted and context-specific response that is characterised by independent yet interdependent activation of cascades of biological responses initiated upon primary injury. These key biological responses, such as excitotoxicity, mitochondrial dysfunction, oxidative stress, delayed cell death onset, and progressive neurodegeneration, all contribute to the augmented damage associated with the primary injury (Borlongan et al. 2015). The diverse nature associated with the secondary injury highlights the complexity of TBI and highlights the various aspects of an injury that can be targeted through therapeutic interventions. The type of primary injury, including blunt vs penetrating, together with the host-specific, delayed secondary molecular response, contribute to the heterogeneity of the injury and highlight the importance of targeting specific pathways and mechanisms relevant to the host’s unique response. This holds the potential for improving the outcomes associated with TBI and is becoming a promising and potential therapeutic avenue through which the pathophysiology of brain injury can be attenuated and treated (McKee & Daneshvar, 2015, Pearn et al. 2017).

To date, no effective therapeutic agent exists for the treatment of TBI. Nevertheless, a large variety of pharmacological therapeutic agents are currently used in the clinical setting with the aim to inhibit the release of pro-inflammatory cytokines, decreasing cellular oxidative stress and the onset of neuronal apoptosis. Practically, however, many agents remain experimental and not part of standard care. These treatments range from calcium channel blockers to NMDA receptor antagonists and steroids, with the aim of inhibiting the pathophysiological cellular hallmarks associated with the secondary injury of TBI (Beauchamp et al., 2008). However, treatment success remains poor, urgently demanding for new approaches to be investigated. One compound that has recently received major attention is antisecretory factor.

Antisecretory factor (AF) was discovered in 1984 by Lönnroth & Lange and is a ubiquitously expressed endogenous protein (Johansson et al. 1997a, b; Hansson et al. 2012a, b). AF16 is present in cells of the pituitary gland, the central nervous system, and the intestinal mucosa (Johansson et al. 1997a, b). The therapeutic role of AF16 was first discovered when it showed potential in the inhibition of intestinal fluid secretion caused by cholera toxin as a combined result of the resistance and desensitisation of the intestinal epithelium to cholera toxin together with the production of an anti-diarrhoeic factor (Lange et al. 1987; Lönnroth & Lange 1984). The anti-secretory motif of this anti-diarrhoeic factor (AF) was extracted from the pituitary glands of piglets using isoelectric focusing and gel chromatography and resulted in a 16 amino acid peptide, named anti-secretory peptide (AF16). AF16 has since been investigated in multiple studies and has proven beneficial in decreasing fluid build-up and inflammation (Al-Olama et al. 2011a, b; Conrady et al. 2013; Clausen et al. 2017a, b; Barrueta Tenhunen et al. 2019), decreasing interstitial fluid as well as decreasing intra-cranial pressure (Al-Olama et al. 2015; Clausen et al. 2017a, b; Cederberg et al. 2020; Gatzinsky et al. 2020). However, the molecular mechanisms through which AF16 exerts protective effects, as well as the context, type, and severity of injury it may operate in, remains poorly understood. Moreover, the intracellular localisation of exogenously administered AF16 has not yet been revealed, further limiting understanding of its molecular mechanisms. Therefore, elucidating the effects of AF16 in the context of the cellular stress response is crucial, and shall be the focus of the present study.

## Materials and Methods

### In Vitro Model

#### Cell Culture Reagents and General Materials

The mouse neuroblastoma cell line, N2A^wt^ cells, were kindly gifted by Professor Sangram Sisodia (Department of Neurobiology, University of Chicago, USA). This model system has been well characterised in the context of neuronal health and disease and was hence chosen for subsequent experimental conduct (Lumkwana et al. 2022; Ntsapi & Loos 2021). Cells were stored in liquid nitrogen and thawed prior to experiments. All cells underwent two passages post-freezing before being seeded for experiments. Following trypsinisation (#25,200,072, ThermoFischer) N2A^wt^ cells were cultured in complete culture media (CCM) consisting of DMEM (#11,965,092, ThermoFischer Scientific) supplemented with 10% FBS (#16,140,071, ThermoFischer Scientific) and 1% PenStrep (#15,140,122, ThermoFischer Scientific) and maintained in a humidified incubator (SL SHEL LAB CO_2_ Humidified Incubator) at 37 °C with a 5% CO_2_ atmosphere. DMEM was added to the cell suspension in a 2:1 ratio to neutralise the trypsin. The cell suspension was then transferred to a 15-mL Falcon tube and centrifuged at 1500 rpm for 3 min at room temperature. The supernatant was removed, and the pellet resuspended in 1 mL of fresh CCM. Cells were then counted using the Countess 3 Automated Cell Counter and seeded at the desired density into flasks and/or appropriate experimental dishes (Supplementary Fig. 2).

### Treatment Conditions and Reagents

To establish a suitable concentration of AF16 peptide (Pieter Siesjõ, Skåne University, Sweden), N2A cells were treated with varying concentrations of AF16 (0.1, 1, 2, 5, 10, and 20 μM) for 24 h after which a WST-1 (CELLPRO-RO, Merck) assay was performed according to the manufacturer’s protocol. In order to assess the protective effects of AF16 on wound closure attributed solely to neuronal migration, cells were treated with 10 µg/mL Mitomycin C (M4287, Sigma). For autophagic flux assessment, cells were treated with 400 nM of Bafilomycin A_1_ (Baf) (B0026, LKT laboratories) for 2 h. After completion of the treatment intervention, cells were either prepared for flow cytometry, microscopy analysis, cell viability assays or harvested for western blot analysis. Primary and secondary antibodies (horse radish peroxidase (HRP) and fluorophore conjugated secondary antibodies) used for western blotting (WB) and immunofluorescence (IF) are listed in supplementary tables as Tables 1 and 2, respectively. Furthermore, fluorescence probes such as LysoTracker Red, MitoTracker Deep Red, NHS-Fluorescein (FITC) (L7528, M2242 and 46,410, ThermoFischer), and Hoechst 33,342 (H6024, Sigma-Aldrich) were used for imaging.

**Table 1:**
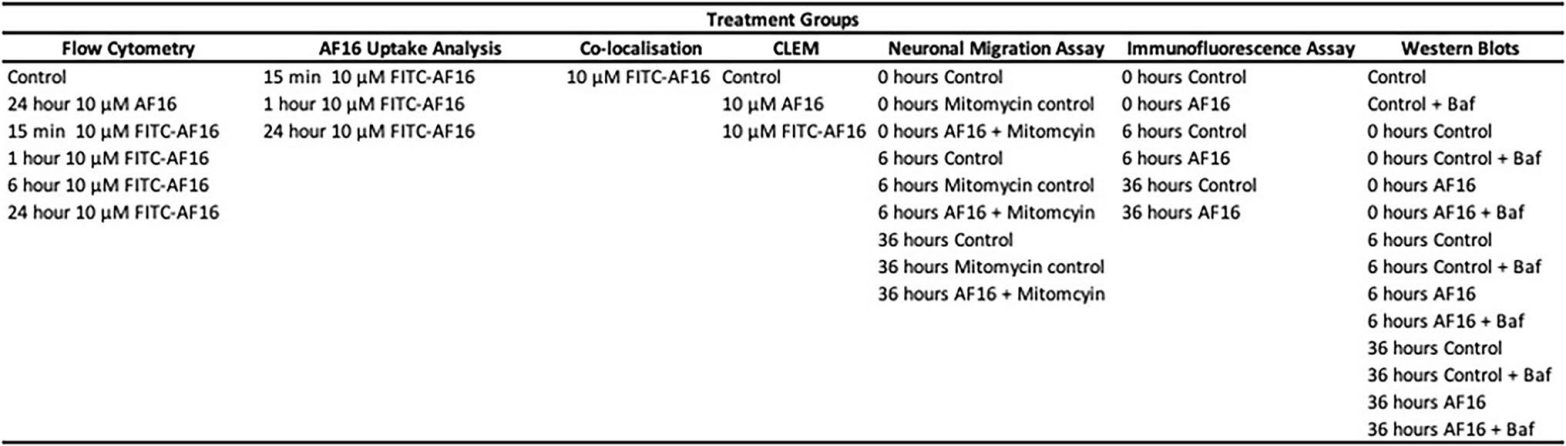
Treatment groups for each respective experiment performed in the study

### NHS-Fluorescein Labelling of AF16

Conjugation of AF16 with FITC allowed for visualisation of the peptide. In brief, FITC was resuspended in DMSO, and AF16 was resuspended in miliQ water (MQ). NHS was added to the AF16 solution and incubated on ice for 2 h. The mixture was loaded onto a C18 Sep-Pak 1 mL column activated with acetonitrile and equilibrated with MQ. The unlabelled NHS was washed from the column using MQ. The FITC-AF16 mixture was eluted from the column using 90% acetonitrile-10% MQ. The FITC-conjugated AF16 was frozen for at least 2 h at − 80 °C after which the sample was freeze-dried.

### Flow Cytometry

In order to assess peptide uptake, N2A^wt^ cells were seeded into T25 flasks, incubated overnight, and treated accordingly (Table 1). At the end of the treatment period, cells were trypsinised and centrifuged. Following trypsinisation, cells were resuspended in 1 mL prewarmed 1X PBS. Cells were then analysed using a BD FACS Melody cell sorter (BD Biosciences), in the FITC channel with a 488-nm laser and 527/32 band-pass filter, after which data obtained was analysed using the BD FlowJo™ v10.6 software. A minimum of 20,000 events was collected and debris and doublets were excluded from the sample analysis. Data was compared to an untreated control. The mean fluorescence intensity was assessed from three independent experiments.

### Confocal Microscopy

All fluorescence microscopy was performed using a Carl Zeiss Confocal LSM 780 Microscope with Elyra PS1 technology.

### Live Cell Imaging

In order to determine the rate at which AF16 is taken up into cells, as well as the intracellular localisation of AF16, peptide uptake was tracked over time. N2A^wt^ cells were seeded into 8 chamber dishes (#155,360, ThermoFisher) and incubated with FITC-AF16 for the respective timepoints (Table 1). Maintaining the cells in the on-stage incubator of the microscope (temperature, CO_2_, and humidity controlled), Z-stacks of each treatment group at respective timepoints were acquired using × 10 and × 63 magnifications. Regions of interest (ROI) were selected using the Icy BioImaging software and Intensity profiles were obtained.

### Staining Procedure for Co-localisation and CLEM

For both co-localisation and correlative light and electron microscopy (CLEM), cells were stained to visualise the nucleus, lysosomes, and mitochondria. Prior to fixation, cells were treated with 60 nM LysoTracker™ Red DND-99 for 30 min, followed by 60 nM MitoTacker™ Deep Red FM for 15 min and 1:200 Hoechst 33,342 for 10 min.

### Co-localisation of FITC-AF16 with Lysosomes and Mitochondria

Cells were seeded into gridded glass bottom culture dishes (P35G-1.5–14-CGRD, MatTek**)** and allowed to incubate overnight. Thereafter, cells were treated with FITC-AF16 for 24 h and stained according to the protocol above. After 24 h of treatment, cells were fixed with 4% PFA for 10 min, washed 3x with PBS, and transferred to the confocal microscope. Z-stacks were acquired using a × 100 magnification and 405-nm, 488-nm, 561-nm, and 633-nm laser excitation. In order to remove any background noise, each Z-stack of a region of interest, obtained through confocal imaging, was separated into its respective colour channels and deconvolved with 20 iterations using an EPIDEMIC plugin available in the Icy Bioimaing software (http://icy.bioimageanalysis.org/plugin/epidemic/). Images were analyzed in ImageJ software for colocalization using the colocalization threshold test.

### Correlative Light and Electron Microscopy (CLEM)

CLEM allows for the localisation, analysis, and evaluation of labelled proteins and organelles of interest at a subcellar ultrastructural level as observed in electron microscopy. This technique serves as a means through which dynamic biological events can be analysed structurally at high resolution. In order to perform CLEM, samples are first prepared for fluorescence microscopy and after acquisition of fluorescence images, the samples are further processed for electron microscopy.

### Sample Preparation and Image Acquisition for Fluorescence Microscopy

N2A^wt^ cells were seeded into a grid patterned MatTek culture dish with a glass bottom (P35G-1.5–14-CGRD**)** and allowed to incubate overnight. Next, cells were treated as follows: a control group, a 10 µM AF16 group, and a 10 $\upmu$ M FITC-AF16 group incubated for 24 h and stained according to the protocol described above. Cells were imaged using a 10X objective and a 3 × 3 tile scan in order to acquire a wide field of view allowing for the visualisation of the grid and allocation of cells of interest. Thereafter, cells of interest were identified within the region of interest, and z-stacks of 30 slices per stack were acquired using × 100 magnification using 405 nm, 488 nm, 578 nm, or 647 nm excitation as well as transmitted light images with TPMT detector, to aid in the accurate overlay of CLEM images. The positions of each imaged region of interest were annotated to ensure accurate trimming of the resin block later during the protocol.

### Confocal Microscopy Image Processing

Images were processed, and fluorescent background and noise were removed using the EPIDEMIC plugin available in the Icy Bioimaging software (http://icy.bioimageanalysis.org/plugin/epidemic/) as before. Deconvolved images were merged with the TPMT channel to be used for accurate overlays.

### Sample Preparation for Electron Tomography

Following acquisition of the fluorescence images, the area of cells imaged was marked at the bottom of the coverslip using a permanent marker. Samples were prepared for electron tomography using a protocol developed by Russell et al. (2017) in which cells were fixed in a mixture containing 2.5% glutaraldehyde and 4% formaldehyde in 0.1 M Sorenson’s phosphate buffer for 30 min at room temperature. Next, cells were incubated for an hour on ice with 2% reduced osmium tetroxide (OsO_4_) consisting of a 1:1 mixture of 4% OsO_4_ and 3% potassium ferricyanide, followed by a 20-min incubation at room temperature with thiocarbohydrazide (TCH), 30-min incubation with aqueous osmium tetroxide and an overnight incubation at 4 °C with 1% uranyl acetate. Between each incubation step, cells were washed 3x with dH_2_O. Next, samples were dehydrated in a graded ethanol series for 5 min each (20%, 50%, 70%, 90%, 100%, and anhydrous 100%). The anhydrous 100% ethanol step was repeated for another 10 min at room temperature, whereafter the sample was incubated with 1:1 mixture of acetonitrile and EPON epoxy resin for 1 h at room temperature. Samples were then incubated at room temperature with 100% EPON for 2 × 90 min. Next, this step was followed by the inversion of a flat bottom capsule filled with 100% EPON onto the demarcated area. The dish, together with the inverted cover slip was incubated for at least 48 h at 60 °C allowing for polymerisation to take place. Once polymerised, the inverted capsule was broken off from the coverslip which now contained the single cells layer embedded in the resin, allowing for clear visualisation of the pattern of the grid using a stereomicroscope. Once the correct grid coordinate was identified, the capsule was trimmed and the resin block face sectioned using a Leica UC7 ultramicrotome system (Leica Microsystems, Austria) and an Ultra 45° 3-mm diamond knife (Diatome US, Hatfield, PA, USA, MS16427) allowing for ultrathin sectioning of 100 nm in thickness (Agar Scientific Ltd, Essex, UK, G3390). All sections from the block face of the sample were collected in order to ensure that all regions of the single cell were captured.

### Electron Microscopy Image Acquisition

Silicon wafers (04AGG3390, Agar Scientific) were mounted onto a 12 mm aluminium SEM specimen stub using carbon conductive double-sided tape (AGG3935, Agar Scientific) and placed inside the chamber of the ThermoFisher Apreo FESEM. Sections were first observed at a low magnification in order to determine the location of cells on the corresponding confocal tile scan. After confirming that the cell locations on the confocal image correspond to that of the electron micrographs, cells on all subsequent sections were imaged to ensure acquisition of multiple Z-depths, thereby increasing the accuracy of the resulting overlays. Sections were scanned at 5 kV accelerating voltage and a 1.6 nA probe current using the T1 trinity detector. Electron micrographs were captured in TIF format at a resolution of 3062 × 2304 pixels.

### Overlay of Fluorescence and Electron Micrographs

Image overlays were performed using the EC-CLEM plugin available in the Icy Bioimaging software (http://icy.bioimageanalysis.org/plugin/ec-CLEM) (Paul-Gilloteaux et al. 2017). This required the identification of a micrograph from the fluorescent confocal image stack which best correlated with the features observed in the SEM image together with the identification of multiple regions of interest overlapping between each image such as the nuclear membrane and outer cell border. Image registration and “warping” were conducted in order to rescale the confocal image to the same pixel array and conformation as that defined in the SEM image allowing for an accurate overlay of the images. A minimum of 30 points were allocated per image registration.

### Neuronal Migration Assay

Neuronal migration assays allow for the evaluation of directed migration and wound closure. This method allows for controlled and repeatable experiments through which the effect of TBI can be monitored in real-time on a cellular level, whilst still allowing for the dissection of the molecular mechanisms underlying the pathophysiological hallmarks associated with TBI. The neuronal migration assay mimics relatively static mechanical systems and has been widely used in studies to assess the secondary injury resulting from TBI as well as for drug testing and discovery in vitro, and therefore was employed in this study (Morrison et al., 1998; Kumaria & Tolias, 2008; Morrison et al., 2011).

Mitomycin C was used to inhibit neuronal proliferation allowing for the observation of neuronal migration following an injury. To obtain a stock solution of 5 mg/mL, 5 mg of Mitomcyin C was dissolved in 1 mL of 1X PBS. The stock solution was aliquoted into tubes and stored at − 20 °C until needed. On the day of use, the stock solution was further diluted in growth media to obtain a desired working concentration of 10 µg/mL. N2A^wt^ cells were seeded into a 24-well and grown to approximately 90% confluency. A sterile 200-µL pipette tip was used to introduce a linear wound in each well. Wells were rinsed with 1 mL CCM to remove debris and replaced with fresh media supplemented with the desired treatment concentrations. Cells were transferred to an EVOS M5000 Imaging System at each respective timepoint. Images were acquired using 4 random regions within the length of the linear wound. The coordinate of each region was marked and used to acquire subsequent images. Images were acquired at time points 0, 6, 12, 24, and 36 h. An image J plugin for the analysis of neuronal migration assays (Wound-healing-size-tool) was used to analyse and measure the wound area at each time point (Suarez-Arnedo et al., 2020). From the area obtained, the percentage and rate of wound closure was calculated using the following formula:

$$\text{Percentage Wound Closure}=\frac{\text{Wound Area}\left(0\text{ hrs}\right)-\text{Wound Area}\left(\text{n hrs}\right)}{\text{Wound Area}\left(0\text{ hrs}\right)}\times 100$$

The rate of wound closure was then calculated by dividing the percentage of wound closure by the time in hours for each group (Liang et al., 2007).

### Scanning Electron Microscopy (SEM) of Neuronal Morphology within Leading Edge of the Wound

N2A^wt^ cells were seeded onto MatTek glass bottom confocal dishes (P35G-1.5–14-C). Cells were allowed to incubate until they reached 90% confluency, after which a linear wound was introduced into each dish using a sterile 200-µL pipette tip. The cell monolayer was rinsed with culture media and replaced with the predetermined treatments. Cells were incubated for 0, 6, and 36 h after which the cells were fixed using a 1:1 mixture of media and 8% PFA for 10 min in an incubator. PFA was removed and cells were washed twice with 1 × PBS. Cells were further fixed in a buffer containing 2.5% glutaraldehyde overnight. The following day cells were washed 2X with 0.1 M NaCacodylate after which cells were incubated with osmium (OsO_4_) for 1 h at 4 °C. The cell monolayer was then washed 3X with mQ-H_2_O after which a series of dehydration steps ranging from 50 to 100% ethanol was performed for 15 min each. Cells were then incubated with 50% hexamethyldisilazane (HMDS) in ethanol for 15 min, followed by 100% HMDS for another 15 min, and allowed to dry overnight in a drying oven. The cover slips were carefully removed from the dishes and mounted onto 12-mm stubs using double-sided carbon tape and double-coated with a thin layer of gold–palladium. The samples were then transferred to a Zeiss Merlin SEM in order to capture images of the surface structure of the cells. Beam conditions during surface analysis were 3 kV and a SE2 detector was used.

### Immunofluorescence

N2A^wt^ cells were seeded into separate confocal dishes and allowed to become 90% confluent. Once confluent a linear wound was introduced into each dish after which culture media was discarded and cells were treated as previously described, followed by fixation for 10 min using 4% PFA. Cells were then rinsed 2X with PBS and permeabilised with 0.1% Triton-X100 for 10 min in the incubator. The cell monolayer was rinsed 2X with PBS before blocking with 3% BSA for 30 min at room temperature. Blocking buffer was removed, cells were rinsed 2X with PBS and cells were probed with primary antibodies (Supplementary Table 1) and allowed to incubate at 4 °C overnight. The following day cells were washed 2X with PBS prior to being incubated with the corresponding secondary antibodies (Supplementary Table 2) for 2 h at room temperature. Cells were counterstained with Hoechst 33,342 (diluted to 1:200 in PBS) for 10 min prior to the final washing step of 3X 5 min washes with PBS. Prior to imaging, Dako® fluorescent mounting media (#S302380, Diagnostech) was added to each dish. Images were acquired using 405 nm, 488 nm, 578 nm, or 647 nm excitation at × 10 magnification allowing for an overview of the injury area as well as at 63X allowing for imaging a magnified region of the injury border. Images were separated into their separate colour channels and a maximum intensity projection (MIP) was performed using Zen 2011 image software. A look-up table (LUT) representing the colour-coded intensity projection of each protein of interest was obtained allowing for the visualisation of intensity versus distance from injury forefront. For quantitative analysis the MIP was transformed into a binarized image allowing for detection of the scratch border and analysis of the change in intensity of the protein of interest relative to the scratch boundary could be obtained (Supplementary Fig. 1).

### Western Blotting

N2A^wt^ cells were seeded into 60-mm petri dishes with 8 mL of growth media per dish. N2A^wt^ cells were allowed to attach to the flask and proliferate until 90% confluency was reached. Three separate linear wounds of similar nature were then introduced into each respective treatment dish. Next, each dish was rinsed 2X with prewarmed PBS followed by incubation with the respective treatment concentrations and timepoints. After the relevant treatments were completed, cells were immediately placed on ice, and culture medium (CM) was aspirated and washed twice with prechilled 1X PBS. Pre-chilled radio immunoprecipitation assay (RIPA) buffer (65 mM Tris Base, 154 mM NaCl, 1% NP-40, 1% Na-deoxycholate, 5 mM EDTA, 5 mM EDTA, 0.1% SDS, 1 mM Na_3_Vo_4_, 1 mM NaF, 1 mM phenylmethylsulphonyl fluoride and protease inhibitor cocktail) was added for 10 min on ice to extract total protein. After 10 min, cells were scraped off the dish using a sterile cell scraper. Lysates were collected in a 2-mL Eppendorf tube, sonicated on ice for 4 s at 5 Hz using a MixSonic (S-4000) (Qsonica), and were left on ice allowing for the foam to subside. The cell lysates were then centrifuged at 8000 rpm at 4 °C for 10 min, whereafter the supernatant was transferred into a new tube and stored at − 80 °C. Protein concentration of the cell lysates was determined using a Bradford Assay. Bradford stock solution was made by dissolving 500 mg Coomassie Brilliant Blue (#27,815, Sigma-Aldrich) in 250 mL 95% ethanol, 500 mL phosphoric acid (#W290017, Sigma-Aldrich) and diluted to 1 L using dH_2_O. The stock solution was diluted to produce a 1:5 working concentration and a protein standard curve was generated by creating a range of BSA concentrations. For the samples, 5 µL of the samples was diluted in 95 µL dH_2_O. Thereafter, 900 µL of Bradford working solution was added to each protein standard or sample tube, vortexed, and allowed to incubate for 5 min in a dark room at room temperature. Absorbance measurements were collected at a wavelength of 595 nm using a Cecil C8 2021 spectrophotometer. A standard curve of protein concentration against absorbance values was obtained, from which the unknown protein concentration of each sample was determined. Next, a working concentration of Laemmli’s buffer (9.09 g Tris, 6 mL 10% SDS, 60 g glycerol, 26,4 g SDS, 0.225 bromophenol blue (B8026, Merck), 225 mL dH_2_O) was prepared by adding 3 parts $\beta$-mercaptoethanol (#444,203, Merck) to 20 parts Laemmli’s loading buffer and mixing thoroughly. The concentration of each protein sample was determined and 15 µg of each sample was added to Laemmli’s buffer in a 2:1 ratio. Samples were boiled at 95 °C for 5 min, centrifuged briefly, and placed on ice.

### Sodium-Dodecyl-Sulphate–Polyacrylamide Gel Electrophoresis (SDS-PAGE) and Western Blot Analysis

Protein samples were separated using a self-cast SDS-PAGE gel consisting of 12% resolving and 4% stacking components. Gels were allowed to set at room temperature and secured using an electrode assembly which was then placed into a Mini-Protean Tetra cell tank (#1658001EDU, BioRad). The buffer chambers were filled with 1X running buffer (10X stock solution diluted to 1:10 with dH_2_O). 3 µL of BLUeye pre-stained protein marker (#94,964, Sigma-Aldrich) was loaded into the first well, followed by the appropriate sample volume in a predetermined order. The electrode assembly was connected to a power supply (Bio-Rad Power Pac 300) and proteins were separated at 100 V for 10 min. Proteins were then transferred to a polyvinylidene difluoride (PVDF) membrane using a Trans-Blot transfer pack (Bio-Rad) and a Trans-Blot turbo transfer system (Bio-Rad). Membranes were blocked for 2 h in 5% fat-free milk made up in TBS-T in order to reduce non-specific binding of antibodies. Membranes were rinsed with TBS-T three times for 3 × 5 min. Thereafter, membranes were incubated overnight at 4 °C in primary antibody (Supplementary Table 1). The following day, membranes were rinsed with TBS-T for 3 × 5 min and incubated for 60 min in the appropriate pre-chilled secondary antibody at room temperature. Membranes were rinsed 3 × 5 min with TBS-T and treated with enhanced chemiluminescence (ECL) (#1,705,061, BioRad) reagent. Protein bands were detected and captured using the ChemiDoc MP System (BioRad). Detected band intensities were quantified and normalized against total protein using the Bio-Rad Image Lab software.

### Statistical Analysis

Results were reported as mean values $\pm$ SEM and statistical analysis was performed using GraphPad Prism v9.4.1 software. One-way ANOVA was employed with a Fisher’s LSD post-hoc test to determine statistical significance. Results were considered statistically significant at a *p*-value < 0.05.

## Results

### Quantification and Visualisation of Intracellular FITC-AF16

**Fig. 1:**
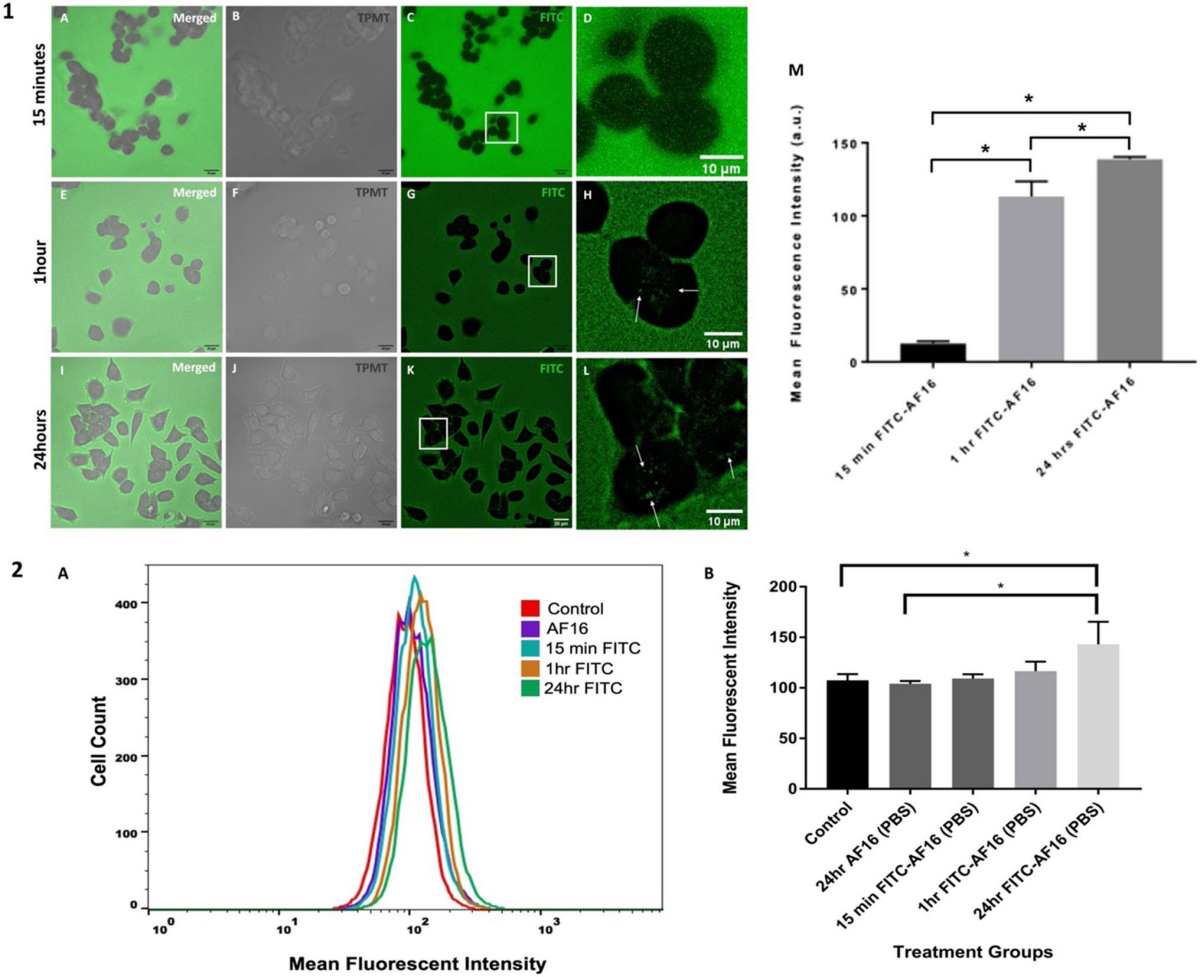
(**1**) Cellular uptake of FITC-AF16 and (**2**) flow cytometry intensity histogram representing the uptake of FITC-AF over time. N2A cells were pre-treated with either FITC-AF16 for 15 min, 1 h or 24 h or with AF16 for 24 h and assessed for mean fluorescence intensity within cells at each respective timepoint using confocal microscopy (**1**) and flow cytometry (2A and B). (**1**) Representative fluorescence images of FITC-AF16 uptake at 15 min, 1 h and 24 h, respectively, presented as the merged image, TPMT channel, FITC-Af16 channel and a zoomed-in region of interest. Quantitative analysis of AF16 uptake is seen in the bar-graph (1 M). More than 10 cells were analysed per *n*, *n* = 4. *p < 0.05 vs 15 min AF16. Scale bar = 20 µm and 10 µm for zoomed in regions. A minimum of 10,000 events per treatment group were acquired and quantitative analysis of mean fluorescence intensity was performed (2B). *n* = 3 and *p < 0.05

To visualise the uptake of the AF16 peptide intracellularly, cells were treated with FITC-AF16 for either 15 min, 1 h, or 24 h and imaged. At 15 min post-treatment, AF16 was detected as a diffused signal throughout the cell (Fig. 1(1)A–D), which changed to more spherical punctate-like structures at 1 h post FITC-AF16 treatment (Fig. 1(1)E–H). At 24 h post-treatment, FITC positive structures were larger in size and brighter in intensity (Fig. 1(1)I–L). Mean fluorescence intensity of FITC-AF16 significantly increased at 1 h [113.2 $\pm$ 10.41] and 24 h [138.7 $\pm$ 1.59] post-treatment when compared to 15 m post-treatment [12 0.56 $\pm$ 1.60 (*p* < 0.05)] (Fig. 1(1)M). Flow cytometry data confirmed the microscopy results, with a significant increase in FITC-signal at the 1 h and 24 h time points compared to untreated control, non-conjugated AF16 treated cells, and 15 min treated cells (Fig. 1(2)A–B).

### Colocalisation of FITC-AF16 with Lysotracker Red and Mitotracker Deep Red Signal

**Fig. 2:**
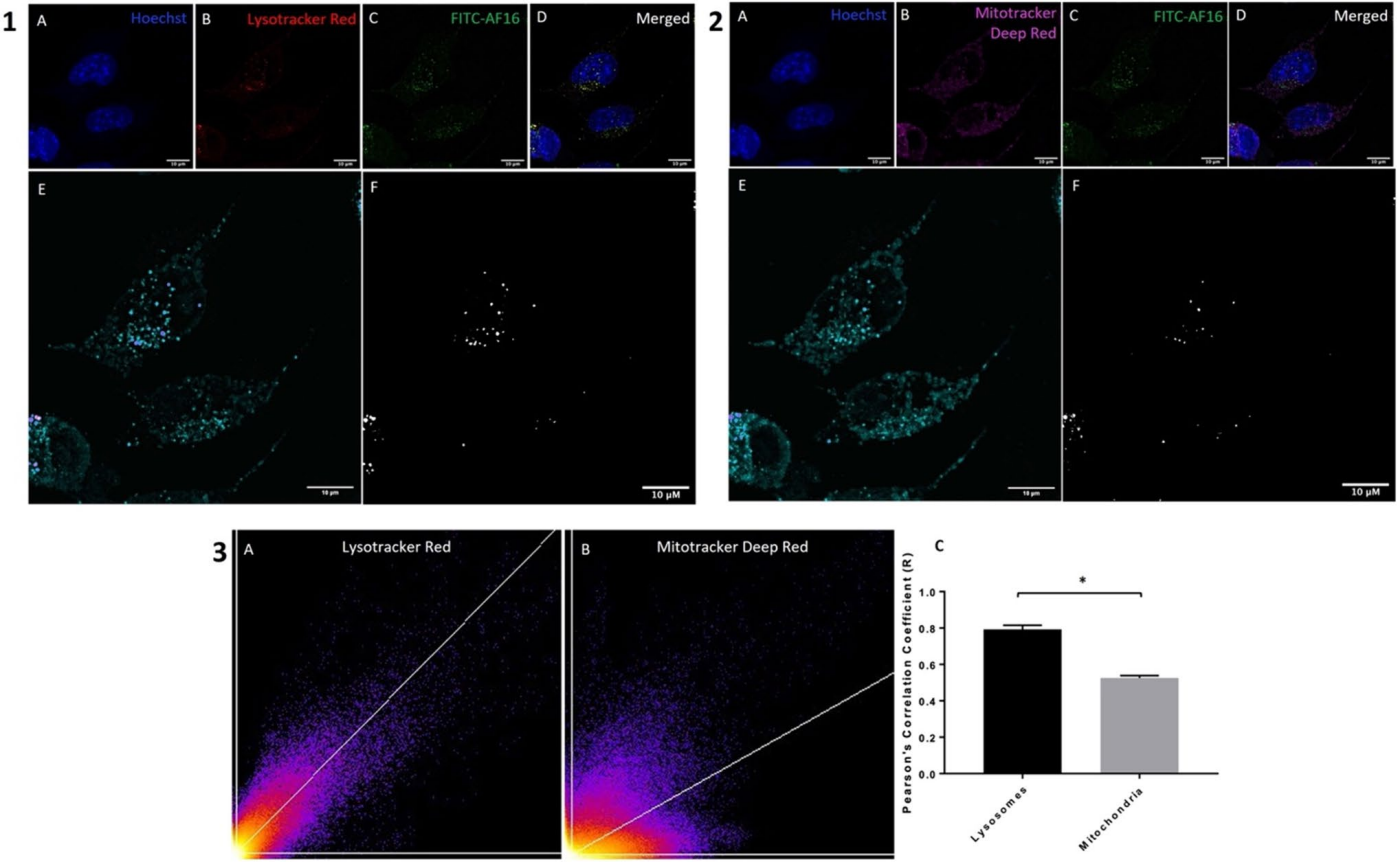
**1** and **2** Representative fluorescence micrographs of colocalisation between FITC-AF16 and (**1**) Mitotracker Deep Red and (**2**) Lysotracker Red. A: nuclei (blue), 1B: mitochondria (magenta), 2B: lysosomes (red) C: AF16 peptide (green), D: merged fluorescent micrograph, E: co-localisation fire LUT, F: co-localised signal only. Scale bar represents 10 µm. **3** Representative scatter diagrams and graphical representation indicating colocalisation

To determine the intracellular co-localisation of FITC-AF16, colocalisation analysis was performed to assess whether FITC-AF16 colocalises with lysosomes and/or mitochondria. A moderate degree of colocalisation was observed between FITC-AF16 signal and Mitotracker Deep Red signal (Fig. 2(2)), with a stronger co-localisation signal observed between FITC-AF16 and Lysotracker Red signal (Fig. 2(1)). Colocalisation metrics revealed a significant increase in the Pearson’s correlation coefficient between FITC signal and Lysotracker Red signal [0.79 $\pm$ 0.02 (*p* < 0.05)] compared to FITC signal and Mitotracker Deep Red signal [0.53 $\pm$ 0.01] (Fig. 2(3)A–C).

### Localisation of AF16 Peptide in N2A Cells Using CLEM

**Fig. 3:**
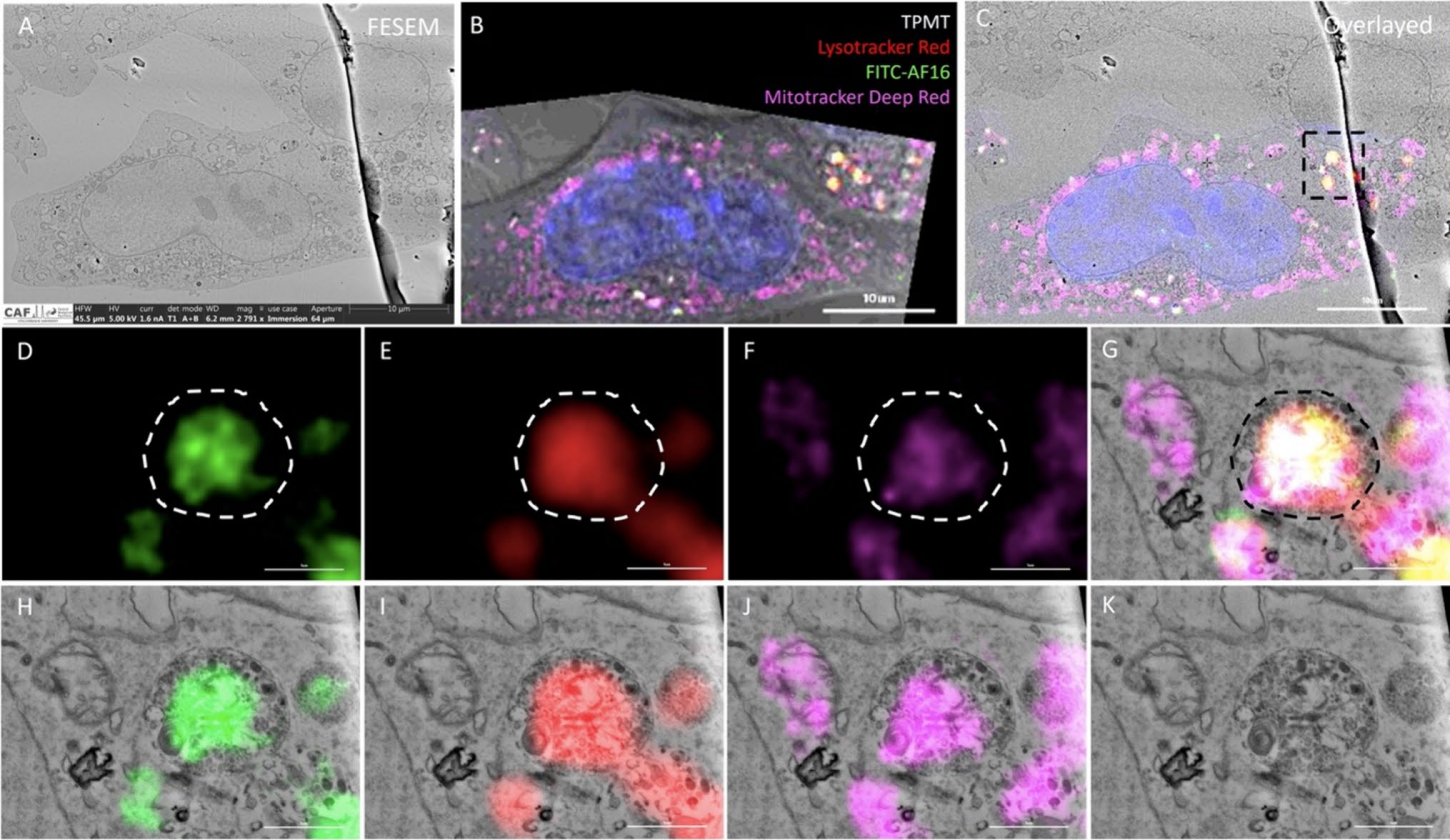
Visualisation and localisation of FITC-AF16 in N2A cells using Correlative Light and Electron Microscopy (CLEM). Representative electron, fluorescence, and overlay micrographs of N2A cells treated with 10 µM FITC-AF16. **A** SEM micrograph of FITC-AF16 treated N2A cell. **B** Confocal fluorescence micrograph of N2A cell at × 100 magnification post image transformation. **C** Overlayed image of the images seen in **A** and **B**. **D** FITC-AF16, **E** Lysotracker Red, **F** Mitotracker Deep Red, and **G** overlayed images of the region of interest outlined in **C**, with the corresponding overlay of these channels displayed in **H**–**J**. **K** FESEM image of the ROI in **C**. White arrowheads indicate FITC-AF16 positive structures and white arrows indicate mitochondria. Scale bar; 10 µm (images **A**–**C**) and 1 µm (images **D**–**K**)

In order to visualise and determine the intracellular localisation of FITC-AF16 within the cell’s ultra-structural context, CLEM was performed (Fig. 3). Cells were co-stained for lysosomes and mitochondria allowing for the confirmation of organelle identification. FITC signal localised within round, double-membraned structures with cargo that appeared homogenous in nature (Fig. 3D–K). When assessing mitochondrial signal, FITC positive double-membraned structures were typically also positive for mitochondrial and lysosomal signal (Fig. 3E, F, G, I, J, K), however not all mitochondria were positive for FITC and/or lysosomal signal (Fig. 3D–K).

### The Effect of AF16 Exposure on the Rate of Wound Closure

**Fig. 4:**
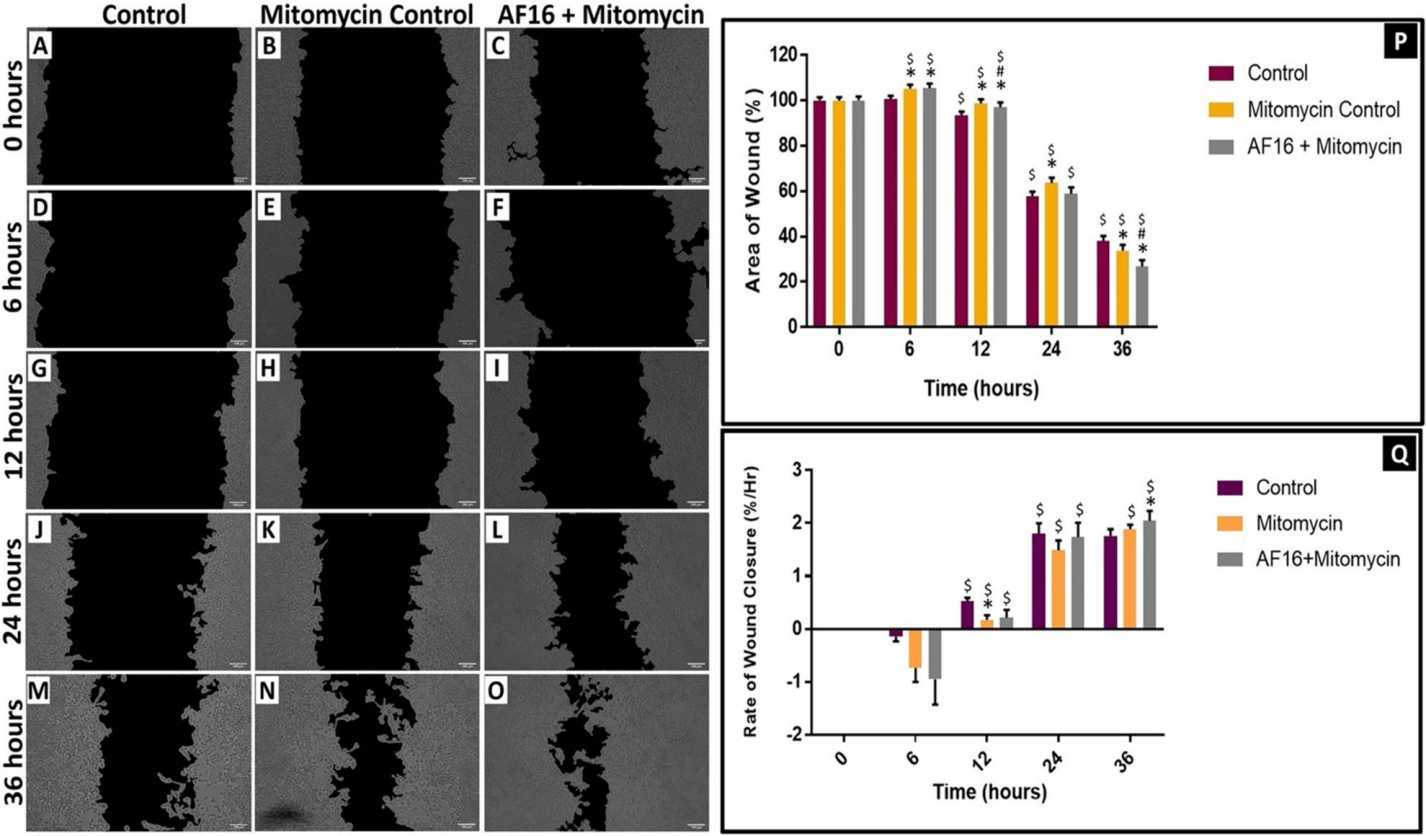
The effect of AF16 treatment over time in an in vitro neuronal migration assay. Representative micrographs of neuronal scratch assay for control (**A**, **D**, **G**, **J**, **M**), mitomycin control (**B**, **E**, **H**, **K**, **N**), and AF16 treated cells (**C**, **F**, **I**, **L**, **O**) are shown. Images were acquired at 0, 6, 12, 24 and 36 h post scratch introduction. Quantitative data of average wound area (*P*) and rate of wound closure (**Q**) are expressed as a percentage of the control (mean $$\pm$$ SEM), *n* = 3 with a total of 4–8 replicates per group. *p < 0.05 vs control at each respective timepoints, #*p* < 0.05 vs Mitomycin control at each respective time point and $*p* < 0.05 vs previous timepoint for each respective treatment. Scale bar = 100 µm

In order to better understand the role of AF16 in a neuronal injury setting, an in vitro 36-h neuronal migration experiment was performed. The average wound area at 6 h was significantly different between control cells [100.6 $\pm$ 1.5% (*p* < 0.05)], mitomycin control cells [105.2 $\pm$ 1.7%], and AF16 treated cells [105.4 $\pm$ 1.99%] (Fig. 4D, E, F). The average wound area at 6 h was larger for each treatment group compared to their respective 0 h time points (Fig. 4P). At 12 h post-injury induction, a significant decrease in wound area was observed in control cells [93.6 $\pm$ 1.6% (*p* < 0.05)], mitomycin control cells [98.7 $\pm$ 1.8%], and AF16 treated cells [97.0 $\pm$ 2.2% (*p* < 0.05)] compared to the previous time point. At 24 h post-injury, the average wound area continued to decrease significantly in mitomycin control cells [63.7 $\pm$ 2.3% (*p* < 0.05)] and AF16 treated cells [58.9 $\pm$ 2.8%] (Fig. 4P), which continued up to the 36 h time point. At 36 h post-injury, the wound area of AF16 treated cells [26.7 $\pm$ 2.9% (*p* < 0.05)] was significantly decreased compared to that of mitomycin control cells [33.9 $\pm$ 2.5%] (Fig. 4P) and compared to the untreated control cells. The rate of wound closure was negative at 6 h post-injury, correlating with the larger wound area at this time point, but significantly increased in control cells [0.4 $\pm$ 0.1%/hr (*p* < 0.05)] and AF16 treated cells [0.2 $\pm$ 0.2%/hr] when compared to mitomycin control cells [− 1.1 $\pm$ 0.3%/hr]. At 36 h post-injury, cells treated with AF16 [2.0 $\pm$ 0.1%/hr (*p* < 0.05)] displayed a significantly increased rate of wound closure compared to both control cells [1.7 $\pm$ 0.1%/hr] and mitomycin control cells [1.8 $\pm$ 0.1%/hr] (Fig. 4Q).

### The Effect of AF16 Exposure on Neuronal Morphology in the Migration Zone

**Fig. 5:**
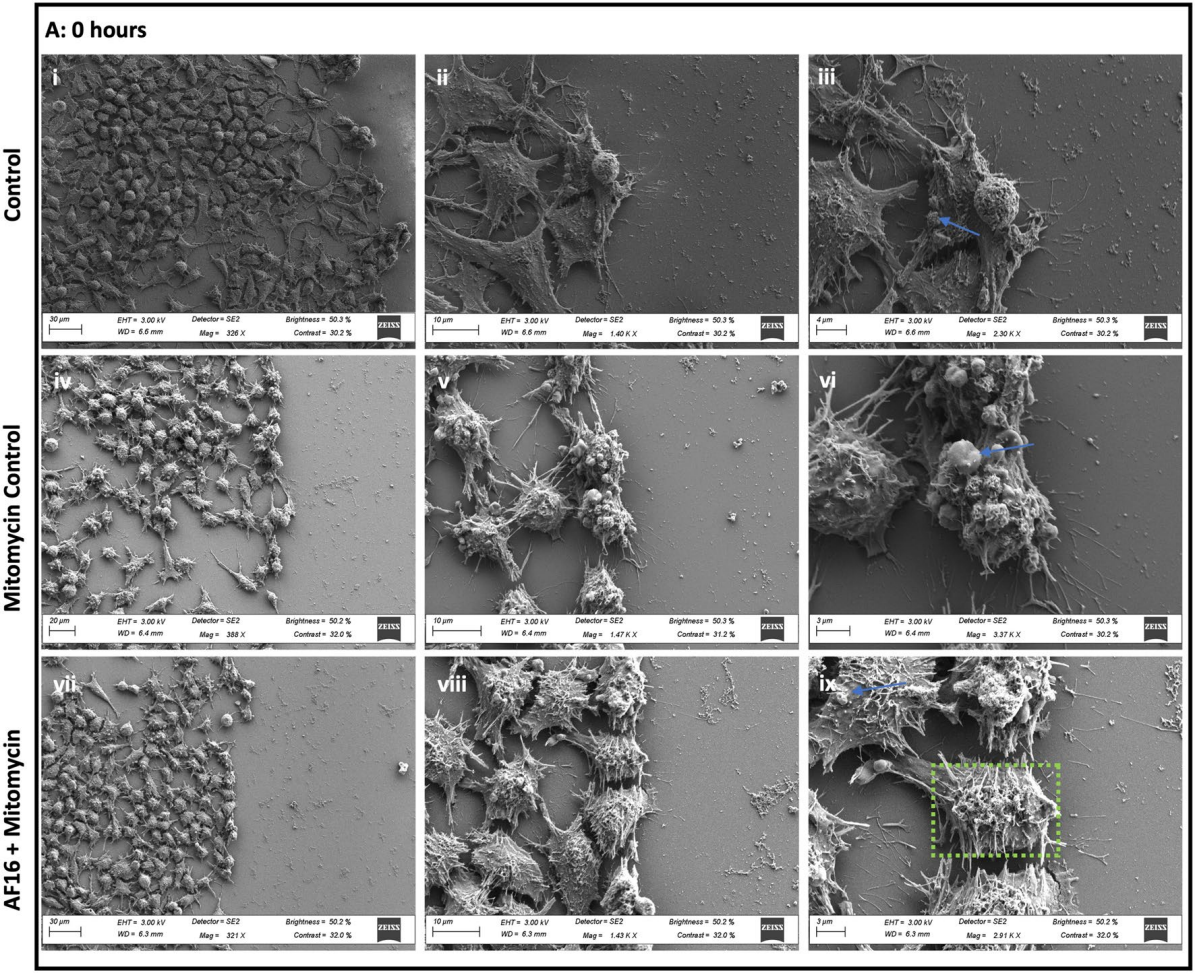

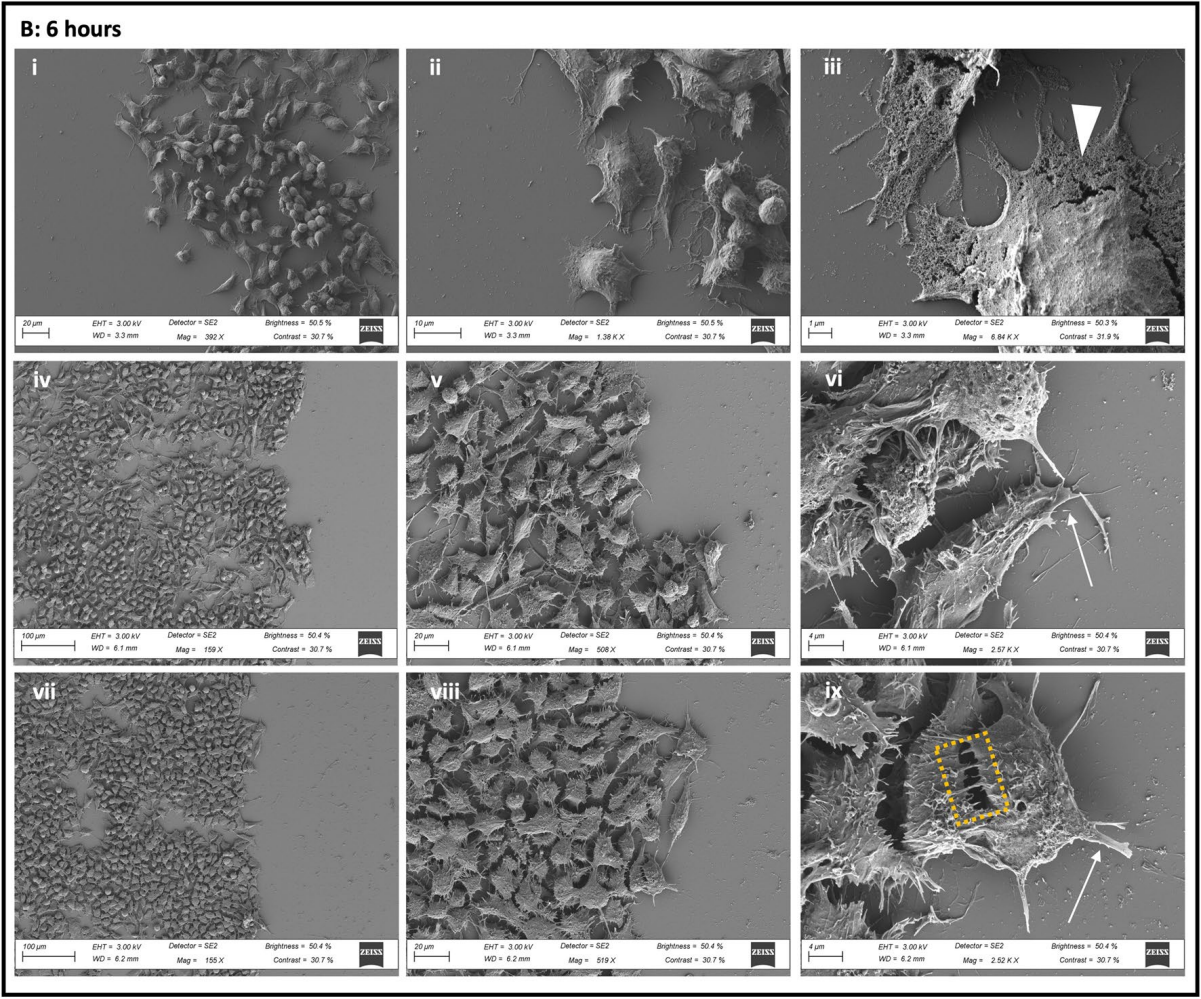

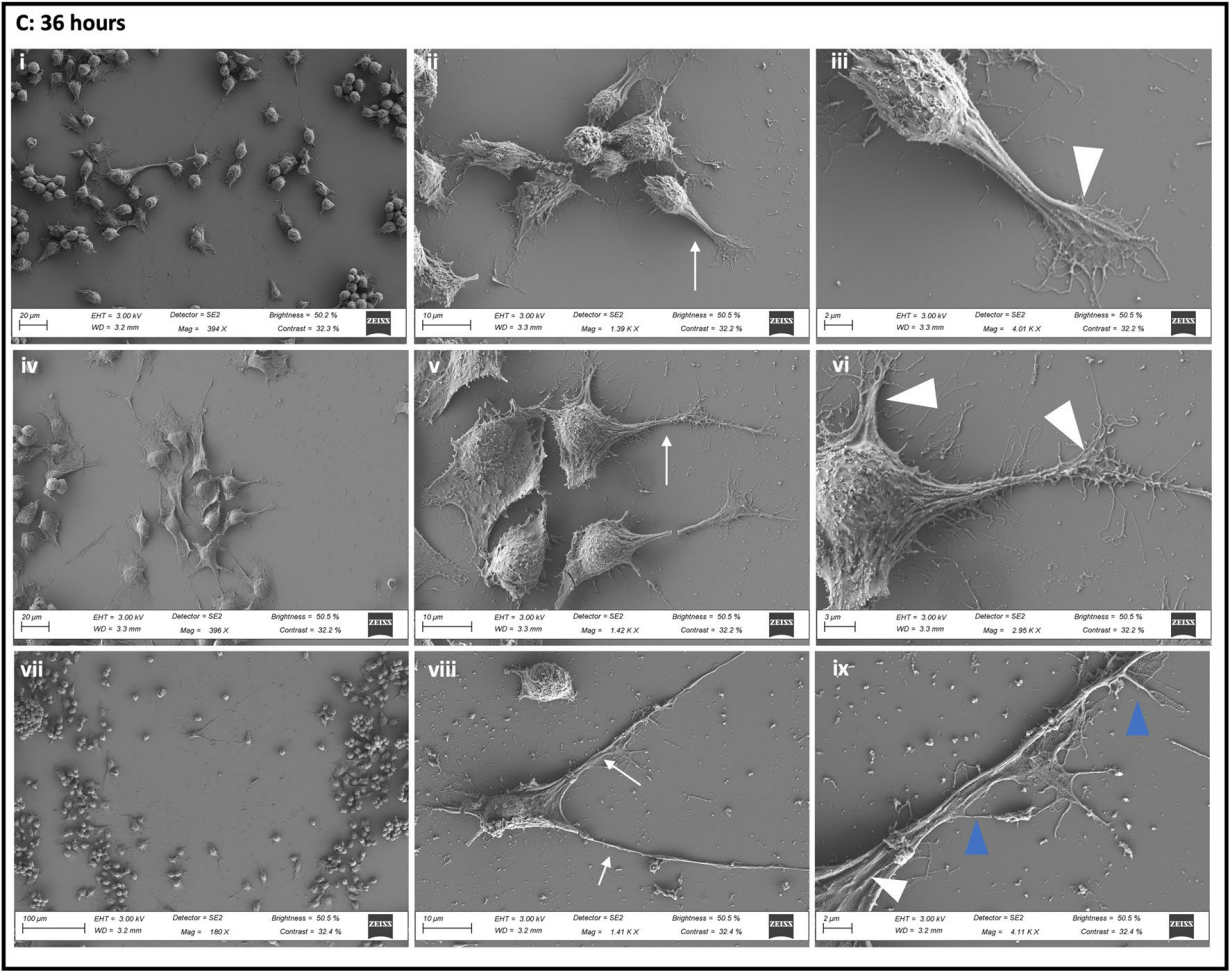
Representative scanning electron micrographs revealing neuronal surface area, surface adhesion and process morphology of cells within the migration zone at 0, 6, and 36 h post-injury. (A: i–iii) Control cells, (A: iv-vi) mitomycin control cells, and (A: vii-ix) AF-16 + mitomycin treated cells. Electron micrographs indicate no leading process formation, cell blebbing (blue arrows), and filamentous microvilli-like extensions on the surface membrane in proximity to the soma (green box). (B: i-iii) Control cells, (B: iv-vi) mitomycin control cells, and (B: vii-ix) AF-16 + mitomycin treated cells. Electron micrographs indicate the presence of flap-like lamellipodia (white arrowheads) with little to no filopodia-like structures and smooth surface membranes in control cells (ii and iii). Mitomycin-treated cells present with a shorter and thicker leading process (white arrow) (iv and v) compared to thinner and longer leading process and increased presence of TNTs (yellow box) in AF16 treated cells (viii and ix). (C: i-iii) Control cells, (C: iv-vi) mitomycin control cells, and (C: vii-ix) AF-16 + mitomycin treated cells. Electron micrographs indicate the presence of lamellipodia (white arrowheads) with filopodia-like structures (blue arrowhead) extending from the leading process (white arrow) (iii, vi, and ix). Control and mitomycin-treated cells present with a shorter and thicker leading process (white arrow) (ii, v) compared to thinner and longer leading process in AF16-treated cells orientated towards the wound (viii). *n* = 3

SEM analysis was performed in order to determine the effect of AF16 treatment on neuronal morphology, process formation and adhesion in orientation towards to wound margin. At 0 h post-injury, no distinct differences were observed between treatment groups, with no leading process being observed within either treatment group, signs of membrane blebbing were observed (Fig. 5A iii, vi). At 6 h post-injury, AF16 treated cells and mitomycin control cells presented with less flap-like lamellipodia together with an increase in the presence of filopodia compared to control cells (Fig. 5B iii, vi, ix). Furthermore, cells treated with AF16 and mitomycin, displayed microvilli-like structures localised on the membrane surface in close proximity to the soma of the cell, compared to the smoother cell surface of control cells (Fig. 5B ix). AF16 treated cells displayed longer and thinner processes as well as longer and more disorganised microvilli-like structures on the plasma membrane compared to mitomycin control cells which presented with thicker process formation and shorter filopodia-like structures. However, at 6 h post-injury, neither treatment groups had any leading processes visible extending exclusively into the wound area (Fig. 5B i, iv, vii). At 36 h post-injury control cells presented with a smoother cell surface compared to that of mitomycin and AF16 treated cells (Fig. 5C ii, v, viii). Both control cells and mitomycin control cells presented with microvilli-like structures on the cell surface membrane as well as filopodia-like structures protruding from the lamellipodia-like leading edge of cells (Fig. 5C iii, vi). AF16-treated cells appeared rounder and presented with clear protruding nuclei when compared to control and mitomycin-treated cells. Importantly, processes within the AF16 treated group tended to be directionally orientated towards the area of injury and were visibly much thinner and longer and appeared filamentous in nature (Fig. 5C ix). Both control and mitomycin control cells displayed thicker lamellipodia-like processes which did not follow any specific orientation in relation to the direction of the leading edge and the wound (Fig. 5C i, iv). Moreover, processes within the AF16 treated group appeared to make contact with other cells in proximity to the wound area (Fig. 5C vii). Furthermore, little to no filopodia like structures were observed within the AF16 treated group, instead well-defined, lamellipodia protrusions extended from the long filamentous processes (Fig. 5C viii, ix).

### Biochemical Analysis and Visual Assessment of Autophagy Pathway Intermediates

**Fig. 6:**
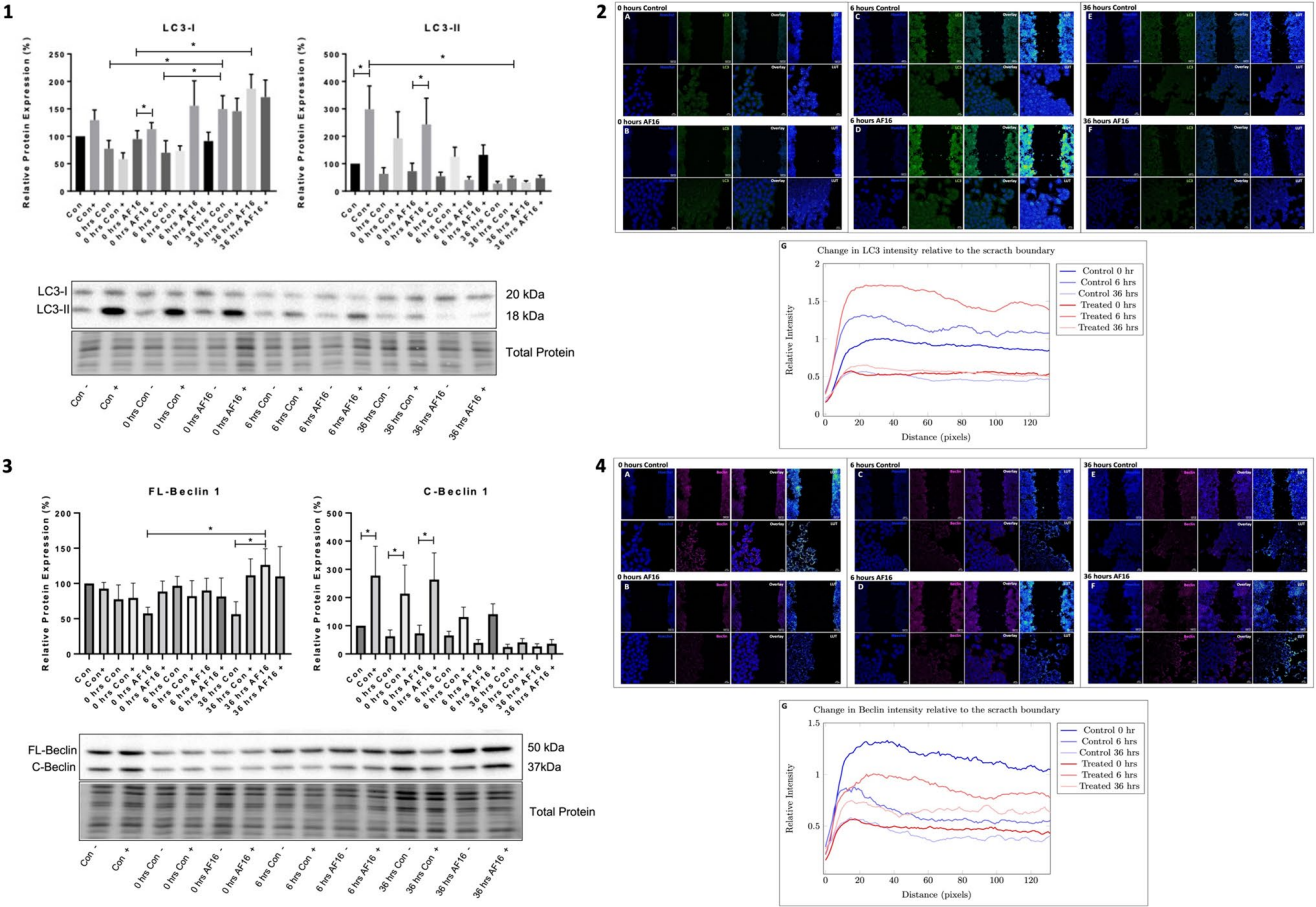
Effect of AF16 exposure on LC3 and Beclin 1 relative protein abundance in the absence and presence of BAF over time together with respective immunofluorescence staining of LC3 and Beclin relative to the wound boundary over time. **1** and **3** Representative immunoblots are shown with treatment groups in the absence and presence of 10 µM AF16 and Baf A1 treated cells at 0, 6, and 36 h post-injury introduction. **2** Representative immunofluorescence images showing the intensity of (2) LC3-II signal and (**4**) Beclin-1 signal relative to the wound area for 0 h (A and B), 6 h (C and D), and 36 h (E and F) post-injury for control and AF16 treated cells respectively. G) Quantitative analysis for the change in intensity distribution profile from the scratch boundary. White boxes indicate regions of interest. *n* = 3. *p < 0.05. Scale bars = 100 µm for top row and 10 µm for bottom row depicting zoomed in regions of interest

To determine whether autophagic activity is altered post-injury, and to assess the effect of AF16 treatment on autophagy pathway intermediates, protein levels of LC3 (Fig. 6(1)) and Beclin-1 (Fig. 6(3)) were assessed using western blot analysis. Furthermore, the abundance of LC3 (Fig. 6(2)) and Beclin (Fig. 6(4)) protein relative to the edge of the wound was visualised and quantified using immunofluorescence.

#### LC3 Protein Expression and Intensity Analysis Relative to the Scratch Boundary

Under normal cellular conditions, N2A cells were characterised by a relatively high basal autophagy flux as a significant increase in autophagosome abundance was observed in Baf treated cells [298.7 ± 85.04%] compared to untreated cells [100 ± 0% (*p* < 0.05)]. At 0 h post-injury a significant increase in LC3-II protein levels was observed for the control [63.0 ± 22.6%] and AF16 [72.6 ± 29.12%] treated cells when compared to their non-Baf treated counterparts [192.7 ± 96.6% (*p* < 0.05)] and [243.1 ± 95.9% (*p* < 0.05)] respectively, indicating a high level of basal autophagy flux. However, at 6 and 36 h post-injury, no significant changes were observed in the presence of Baf for either control or AF16-treated cells when compared to their non-Baf-treated counterparts, indicating a decrease in the rate of degradation in both conditions (Fig. 6(1)). It is interesting to note, that although no significance was observed, an increase in trend in LC3-I protein expression over time is observed for both treatment groups; however, a slightly higher increase is observed in AF6 treated cells. The overall intensity of LC3 signal in both groups over time increased at 6 h post-injury and decreased at 36 h (Fig. 6(2)C–F). When comparing the intensity of LC3 at the scratch boundary between groups, LC3 signal appeared highest at 6 h post-injury in AF16-treated cells (Fig. 6(2)D).

#### Beclin-1 Protein Expression Levels and Intensity Analysis Relative to the Scratch Boundary

When assessing Beclin protein expression, a significant increase in FL-Beclin was observed between 36 h AF16 treated cells [126.4 ± 22.87%] and 36 h control cells [56.43 ± 17.7% (*p* < 0.05)] (Fig. 6(3)). The overall Beclin intensity in the injury zone was higher in AF16 treated cells at 6 and 36 h post-injury compared to control cells, with intensity decreasing along the distance away from the scratch (Fig. 6(4)A–F).

### Biochemical Analysis and Visual Assessment of Mitochondrial Fission and Fusion Proteins and a Marker of Mitochondrial Oxidative Stress

**Fig. 7:**
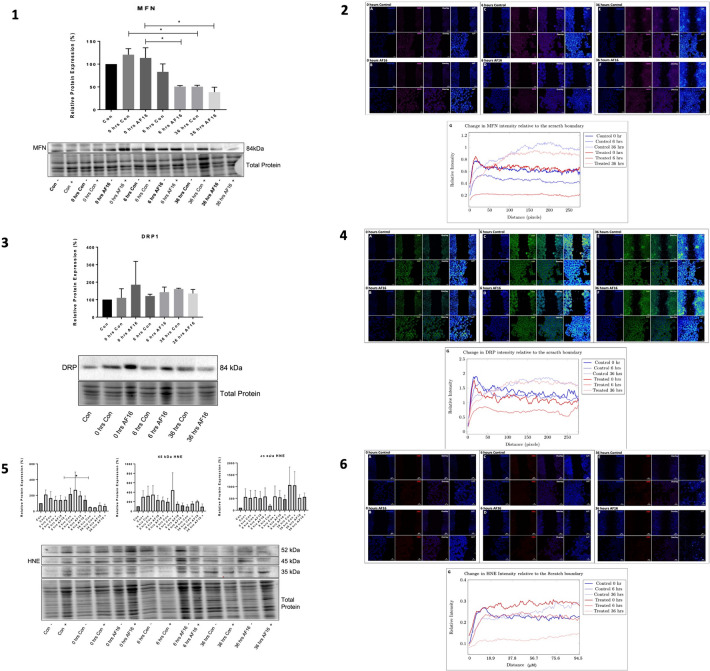
Effect of AF16 exposure on mitochondrial dynamic proteins (**1**) MFN1, (**3**) DRP1, and (**5**) HNE relative protein abundance over time together with respective Immunofluorescence staining of mitochondrial proteins relative to the wound boundary over time. (1, 3, and 5) Representative immunoblots are shown with treatment groups in the absence and presence of 10 µM AF16 and Baf A1 treated cells at 0, 6, and 36 h post-injury introduction. (2, 4, and 6) Representative immunofluorescence images showing the intensity of (**2**) MFN signal, (**4**) DRP signal, and (**6**) HNE signal relative to the wound area for 0 h (A and B), 6 h (C and D), and 36 h (E and F) post-injury for control and AF16 treated cells, respectively. G) Quantitative analysis for the change in intensity distribution profile from the scratch boundary. White boxes indicate regions of interest. *n* = 3. *p < 0.05. Scale bars = 100 µm for top row and 10 µm for bottom row zoomed in regions of interest

To determine the effect of AF16 exposure on mitochondrial dynamics, fission, fusion, and oxidative stress proteins were assessed using western blot analysis (Fig. 7(1), (3), (5)). In order to determine the localisation and change in these proteins relative to the scratch boundary, immunofluorescence analysis was performed at 0, 6, and 36 h post-injury (Fig. 7(2), (4), (6)).

#### MFN Protein Expression Levels and Intensity Analysis Relative to the Scratch Boundary

When assessing MFN1 protein expression, a significant decrease in MFN1 was observed between 0 h [113.6 ± 22.24%] and 6 h [50.69 ± 2.16% (*p* < 0.05)] post-injury (Fig. 7(1)). The overall intensity, which represents the relative abundance of MFN1 present within cells, increased gradually between 0 and 36 h in both control cells and AF16-treated cells (Fig. 7(2)). MFN1 intensity decreased with the distance away from the scratch boundary for 0 and 6 h; however, for 36 h, it increased substantially.

#### DRP Protein Expression Levels and Intensity Analysis Relative to the Scratch Boundary

No significant changes in DRP1 expression were observed for either treatment group over time (Fig. 7(3)). Furthermore, the relative intensity of DRP1 was higher in control cells than AF16 treated cells at 0 and 6 h post-injury, with a similar intensity profile at 36 h post-injury (Fig. 7(4)A–F).

#### 4-HNE Intensity Analysis Relative to the Scratch Boundary

When looking at the overall expression of 4-HNE, no significant differences of note were observed; however, a trend towards lower HNE expression is observed in AF16-treated cells compared to control cells (Fig. 7(5)). At 36 h post-injury, control cells presented with a significantly higher relative intensity of 4HNE than AF16-treated cells (Fig. 7(6)). Furthermore, 4HNE signal at the 36 h post-injury time point appeared to reside within the nucleus, with control cells presenting with an increased number of cells positive for nuclear signal compared to AF16 treated cells (Fig. 7(6)C, F).

## Discussion

TBI is referred to as a silent epidemic (Dewan et al. 2019). TBI has both a primary injury component, as well as a delayed secondary injury component which in certain cases is manageable through medical interventions (Khellaf et al. 2019). The secondary brain injury is associated with excitotoxicity, increased ROS production, mitochondrial swelling and dysfunction, oxidative stress, fluid build-up, and increased ICP (Werner & Engelhard 2007). Despite the global impact of TBI, failure in establishing an effective treatment intervention remains a major problem. AF16 has previously shown promising protective effects in cases of diarrhoea (Lonnroth et al. 2003; Nicolas and Moal 2015; Zaman et al. 2018), cancer (Ilkhanizadeh et al. 2018a, b, c; Lange et al. 2020), and inflammation and fluid build-up (Al-Olama et al. 2011a, b; Conrady et al. 2013; Clausen et al. 2017a, b; Barrueta Tenhunen et al. 2019). As a result of this, AF16 peptide has recently received increasing attention as a possible treatment for TBI, especially due to the fact that AF16 has shown promise in attempts to decrease ICP in both experimental in vitro and in vivo models of TBI (Al-Olama et al. 2015; Clausen et al. 2017a, b; Cederberg et al. 2020; Gatzinsky et al. 2020). However, the intracellular localisation of AF16 as well as the underlying molecular mechanisms through which AF16 exerts its protection are largely unknown. Therefore, we firstly aimed to characterise the uptake of AF16 into cells over a period of 24 h. In order to achieve this, cells were treated with fluorophore tagged FITC-AF16 to allow for the visualisation and uptake quantification of AF16 using confocal microscopy and flow cytometry. To determine the precise intracellular localisation of of AF16 in the context of key organelles of interest, we performed colocalisation of FITC-AF16 with mitochondria and lysosomes. Moreover, CLEM analysis was used to visualise FITC-AF16 within its ultrastructural context as well as to elucidate its effects on the ultrastructure of organelles critical in the maintenance of cell viability, such as mitochondria and lysosomes. In the second part of the study, we investigated the effect of AF16 using a model of neuronal injury. By performing a neuronal migration scratch assay, we evaluated the directed migration, followed by SEM for the analysis of process formation and adhesion of neurons within the leading edge of the wound. Lastly, to better understand the molecular mechanisms through which AF16 may exert its protective effects, western blot analysis was performed for key autophagy*-* mitochondrial- and oxidative stress- markers such as LC3, Beclin1, MFN1, DRP1, and 4HNE respectively.

### FITC-AF16 Uptake Results in Distinct Cytoplasmic Signal Hotspots

Our viability assay indicates that the chosen concentration for AF16 was non-toxic to N2A cells as no change in reductive capacity was observed following treatment with AF16 (Supplementary Fig. 2). A concentration of 10 µM was chosen for all subsequent experiments, guided by these results and literature (Jennische et al. 2008; Al-Olama et al. 2011a, b; Hansson et al. 2012a, b; Clausen et al. 2017a, b; Bazzurro et al. 2018; Ilkhanizadeh et al. 2018a, b, c). Treatment of cells with FITC-AF16 revealed that over 24 h, cells showed a significant increase in FITC-AF16 uptake (Fig. 1(1)M and Fig. 1(2)B) which presented as diffused signal 15 min post-administration (Fig. 1(1)D), thereafter adjusting to a signal pattern, with structures becoming stronger in signal and larger in size over time (Fig. 1(1)H, L). To our knowledge, exogenous treatment of AF16 and its time dependent uptake in neuronal cells have not previously been investigated visually. However, it is known that cells endogenously express AF16 (Davidson & Hickey 2004). Upon exogenous administration of tagged AF16, a similar characteristic punctate pattern of AF16 uptake was observed by Dzebo (2014) as well as Ilkhanizadeh et al., (2018a, b, c). Despite the fact that a different fluorescent tag was used, as well as a non-neuronal cell line, these findings support that which we observed. This distinct punctate staining pattern within the cytoplasm is typically characteristic for molecules taken up through endocytosis (Matson Dzebo 2014), suggesting that AF16 may be taken up through similar mechanisms.

### FITC-AF16 Localises to Lysosomes

Observations associated with the uptake of endogenously administered AF16, preceded and gave rise to a need for colocalisation analysis to investigate the nature of the intracellular localisation of AF16 peptide. Given the vital role of mitochondria and lysosomes in preserving cell viability through means of maintaining cellular homeostasis, these organelles were chosen as a point of departure. A moderate degree of colocalisation was observed between FITC-AF16 signal and Mitotracker Deep Red signal (Fig. 2(2) and Fig. 2(3)B, C), however significantly higher co-localisation was observed between FITC-AF16 signal and Lysotracker Red signal (Fig. 2(2) and Fig. 2(3)A, C). Although intracellular localisation of AF16 has been shown, no literature exists regarding the colocalisation of AF16 with organelles. A relationship exists between lysosomes and the endosomal pathway with an important function of lysosomes in the digestion of exogenous material taken up through means of the endosomal pathway (Cooper 2000). Therefore, the colocalisation between lysosomal signal and FITC-AF16 signal substantiates the notion of the possibility of AF16 uptake intracellularly through the endosomal pathway. Furthermore, the exact localisation of co-localised structures was observed predominantly in the perinuclear region (Fig. 2(1)E, F and Fig. 2(2)E, F). Lysosomes operate in close context with autophagosome synthesis and autophagosome-lysosome fusion and change their position intracellularly in response to nutrient availability (Korolchuk et al. 2011; Raiborg 2018), with a perinuclear lysosomal clustering pattern seen under conditions of increased autophagy activity. Due to the perinuclear localisation of lysosomes in cells treated with AF16 observed in Fig. 2(1)E and F and Fig. 2(2)E and F, it is plausible that lysosomal activity is increased upon AF16 treatment. Lysosomes are vital in the regulation of autophagy activity. Sarkar et al., (2014) have shown that the accumulation of autophagosomes following TBI, resulted from dysfunctional lysosomes. Together with their indispensable role in macro-autophagy, lysosomes are also vital for the regulation of micro-autophagy, which requires the engulfment of cytoplasmic content through direct lysosomal engulfment (Park et al. 2015; Yim & Mizushima 2020). Therefore, the possible increase in lysosomal activity upon AF16 exposure may aid in regulating autophagy.

### AF16 Localises to the Autophagy Compartment and Impacts Mitophagy

CLEM was employed to visualise and localise the intracellular signal of FITC-AF16 within the cellular ultrastructural context. Our results revealed that FITC-AF16 localises to double-membraned vacuoles characteristic of autophagosomes (Fig. 3D, G, H, K) (Eskelinen 2008). To our surprise, some of these FITC-AF16 positive autophagy vacuoles also stained positive for Lysotracker Red signal as well as Mitotracker Deep Red signal (Fig. 3 D–J), suggesting a role for autolysosomes as well as the degradation of mitochondria through autophagy, suggesting the possibility of mitophagy. To our knowledge, this is the first study in which intracellular localisation of AF16 and its effect on organelle ultrastructure are assessed. Our results suggest the possible involvement of AF16 on the autophagy-lysosomal system, as well as a component of mitophagy as a means for mitochondrial quality control. It is known that colocalisation of mitochondrial signal with lysosomal signal is used as a means to assess mitophagy (Ding & Yin 2012; Dolman et al. 2013; Williams et al. 2017), supporting the notion of mitophagy regulation upon AF16 exposure.

Next, in order to better reveal the effect of AF16 on mitophagy, colocalisation analysis between mitochondrial and lysosomal signal was performed. Control cells presented with a higher degree of mitochondrial and lysosomal signal colocalisation (Supplementary Fig. 5 E, F, G, I, J) when compared to AF16 treated cells (Supplementary Fig. 6 E, F, G, I, J) which presented with a stronger mitochondrial signal than lysosomal signal together with a decrease in colocalised signal. Colocalisation of mitochondrial and lysosomal signal is indicative of mitophagy, therefore, due to the decrease in co-localisation between these signals upon AF16 treatment, we speculate that AF16 exerts a protective effect by decreasing the number of mitochondria degraded through the mitophagy pathway. The increased rate of mitophagy in neuronal cells can thus be attributed to the increased removal of dysfunctional cells which is critical for the maintenance of neuronal health (Evans & Holzbaur 2020). A decrease in mitophagy would therefore increase the mitochondrial pool, which may impact cell metabolism and energetic charge. Furthermore, of note is the observation of round electron dense material within the endoplasmic reticulum of AF16 treated cells (Supplementary Fig. 4B3) which is not observed in control cells. The endoplasmic reticulum is well known for its role in protein synthesis, as well as the ER response (Schwarz & Blower 2016), and due to the peptide nature of AF16, it may be plausible that the exogenous administration of AF16 impacts the ER stress response resulting in the subsequent upregulation of survival protein synthesis.

### The Effect of AF16 on Neuronal Migration

In the second part of this study, we aimed to determine whether AF16 plays a protective role in a model of neuronal injury. Scratch wound assays are widely used to mimic the injury response observed following a trauma event. The injury caused by a scratch wound assay is, in many aspects, similar to that observed in TBI, such as mechanical damage, severing of cell processes and membrane rupture. Furthermore, cells are exposed to chemical cues released by adjacent dying and/or dead cells (Jowers et al*.,* 2013).

### AF16 Exposure Accelerates Wound Closure

Our results revealed that AF16 exposure resulted in a significant decrease in the wound area (Fig. 4P) and an increase in rate of wound closure (Fig. 4Q) over time compared to control cells. Of note, degeneration of cells within the wound area at 6 h post-injury was observed (Fig. 4D–F), resulting in an increased wound area compared to the respective 0 h time points. This degeneration was significantly higher in AF16-treated cells than in control cells (Fig. 4D, F, P). The degeneration of neuronal extensions closest to the wound border has previously been shown by Lööv et al., (2013) however, the reason for neuronal degeneration prior to neurite regeneration is unknown. Although scratch wound assays have previously been used to analyse the effects of specific treatment interventions in the context of TBI (Jowers et al*.,* 2013; Parmientier-Batteur et al*.,* 2011; Sun et al*.,* 2020), no previous research has been conducted investigating the effect of AF16 on wound closure or its effect on neuronal morphology within the wound area. Our results provide evidence that AF16 enhances the overall wound healing capacity of neurons and exerts protective effects as early as 6 h post-injury. Moreover, an increased regeneration of neurons (Fig. 4G–O) followed by an increase in the rate of neuronal migration (Fig. 4P) and process length post-injury (Fig. 5) compared to control cells was revealed.

### AF16 Exposure Leads to Increased Length of Neuronal Processes and Decrease in Filopodia

Upon further inspection of ultrastructural surface morphology, when comparing AF16 treated cells to that of control cells, SEM image data revealed the presence of additional features such as increase in the length of processes in AF16 treated cells compared to control cells (Fig. 5). These processes presented with few filopodia-like structures, however flap-like lamellipodia-like structures were observed along the length of the process (Fig. 5A, B, C vii-ix). Control cells on the other hand presented with somewhat thicker and shorter processes characterised by an increase in filopodia-like side protrusions (Fig. 5A, B, C i-iii). Lamellipodia are meshwork bundles of actin filaments which constitute a major force contributing to cell movement, whereas filopodia control the directionality of cell migration (Bornschlögl 2013; Innocenti 2018; Leithner et al. 2016; Mattila & Lappalainen 2008). Lamellipodia on the other hand are known to present with weak adhesion forces compared to filopodia which promote adhesion in order to stabilise the lamellipodium (Giannone et al. 2007). It has been shown that an increase in the presence of filopodia results in slower migration of cells, whereas lamellipodia are involved in faster cell migration (Innocenti 2018; Leithner et al. 2016), supporting the notion that AF16 may increase the rate of cell migration whilst resulting in a decrease in cell adhesion.

### AF16 Exposure Results in an Increase in Autophagosome Synthesis

Autophagy is a key degradative pathway through which cellular homeostasis is maintained (Wang & Klionsky 2003); however, its role upon exposure to AF16 within neuronal injury, has not previously been investigated. Assessing the abundance of LC3-II protein expression (Fig. 6.1), our results revealed a high basal autophagic flux for N2A cells under control conditions. Upon introduction of neuronal injury, decreased LC3-II expression over 36 h was observed for both control and AF16-treated cells. Furthermore, our results revealed that initially cells displayed an increased level of autophagy flux due to increased presence of autophagosome accumulation in the presence of Baf treatment (Fig. 6.1). Autophagy flux decreased over time for both treatment groups. These findings suggest that neuronal injury results in a decrease in LC3-II protein levels and autophagy flux over time (Fig. 6.1) equally for both control and AF16-treated cells, suggesting no impact of AF16. No coherent findings regarding the effect of TBI on relative LC3-II protein expression exists, with many conflicting results observed in various studies ranging from an increase in LC3-II protein expression caused by TBI over time (Gomez & Clarke 2007; Lai et al. 2008; C. L. Luo et al. 2011), to a decrease in LC3-II protein expression over time (Batulu et al. 2019; Zeng et al. 2020). The changes observed in LC3-II expression could likely be attributed to the magnitude and severity of the injury.

Beclin-1 is a key autophagy protein responsible for the activation of autophagy; however, it has previously been shown that Beclin-1, when cleaved by caspases, negatively regulates autophagy resulting in neuronal cell loss through apoptosis (Luo and Rubinsztein 2010; Bieri et al. 2018). We therefore assessed full-length Beclin-1 (FL-Beclin) which regulates autophagy, as well as the C-terminal Beclin-1 (C-Beclin) which activates apoptosis. Our results revealed an increase in FL-Beclin upon AF16 exposure over time (Fig. 6.3). Furthermore, an increased expression of FL-Beclin was observed in AF16 cells at 36 h post-injury when compared to control cells (Fig. 6.3). This increase in Beclin protein expression was also observed by Luo et al., (2011) and Bao et al*.,* (2015) and is indicative of an increase in autophagy initiation. Next, we assessed the relative protein expression of C-Beclin. In control cells and AF16-treated cells a decrease in C-Beclin was observed over time with a slightly lower expression within AF16-treated cells, albeit not significantly (Fig. 6.3). Taken together, the markers of autophagy assessed, suggest a decrease in autophagy flux following neuronal injury under control conditions (Fig. 6.1 and Fig. 6.3). FL-Beclin protein levels indicate a decreased protein expression over time, further suggesting a decrease in autophagy induction (Fig. 6.3). Subsequently, for cells treated with AF16, a similar decrease in autophagy flux was observed over time, with an increased FL-Beclin expression over time. Our results therefore suggest that AF16 may trigger autophagy initiation. However, downstream effects which result in the decreased autophagy flux observed, are yet to be elucidated.

### AF16 Increases Markers of Autophagy in the Immediate Region of Injury

When assessing intensity of autophagy substrates in proximity to the wound area (Fig. 6.2 A–F) it was observed that LC3 signal intensity was at its highest closest to the immediate region of the injury at 6 h post-injury and decreases with increasing distance from the injury region, with a markedly higher signal intensity observed in AF16-treated cells (Fig. 6.2 B, E). A similar intensity profile was observed for Beclin fluorescence intensity, with an increased signal intensity observed along the wound boundary at 6 h for AF16 treated cells compared to control cells (Fig. 6.4 B, E). These findings support the notion of an increase in autophagy activity within the wound area, in cells treated with AF16. The upregulation of autophagy activity as early as 6 h post-injury in AF16-treated cells suggests a role of autophagy as part of a stress response, which may confer enhanced protection within a neuronal injury setting. Therefore, although overall LC3-II levels post-injury, based on whole protein lysate, are decreased upon AF16 administration, autophagy activity appears to be upregulated along the immediate region of the injury as early as 6 h post-injury, when compared to control cells (Fig. 6.2 A, B). It is well known that autophagy is activated post TBI (Zhang & Wang 2018), with a further increase in autophagy activity through treatment with Rapamycin, resulting in a decrease in brain injury (Carloni et al. 2008). Furthermore, Gao et al*.,* (2016) and He et al., (2018) observed that when enhancing autophagy activity, a decrease in apoptosis as well as an increase in neurobehavior was observed. However, excessive autophagy activation has also been shown to result in the induction of cell death subroutines (Feng et al. 2017). Therefore, the resulting upregulation of LC3-II upon AF16 exposure, within the injury region at 6 h post-injury with a decrease in intensity at 36 h post-injury may confer protection through the upregulation of autophagy, however, not to the extent of excessive autophagy activity.

### AF16 Inhibits Mitochondrial Fission Post-Injury

Mitofusin 1 (MFN) is widely known to initiate fusion of individual mitochondria through outer membrane fusion (Koshiba et al. 2004), whilst Dynamin-related protein 1 (DRP) mediates mitochondrial fission (Smirnova et al. 2001). It is well known that TBI disrupts mitochondrial bioenergetics and results in the accumulation of dysfunctional mitochondria; however, comprehensive understanding regarding mitochondrial dynamics following TBI is lacking (Di Pietro et al. 2017; Simmons et al. 2020). MFN and DRP protein levels were analysed so as to investigate the role a neuronal injury on mitochondrial dynamics and to assess whether AF16 is involved in preserving mitochondrial dynamics post-injury. MFN protein levels increased upon the introduction of injury after which MFN protein expression significantly decreased over 36 h for both experimental groups compared to control cells (Fig. 7.1). Treatment with AF16 resulted in no significant change in DRP protein expression compared to non-treated cells (Fig. 7.3).

When investigating mitochondrial fission through the assessment of relative DRP protein expression post-injury over time, our results revealed a tendency towards increase in DRP protein expression for control cells, however upon exposure to AF16, DRP1 proteins levels remained relatively constant (Fig. 7.3). To our knowledge, few studies have assessed the role of MFN1 expression within a neuronal wound injury model; however, MFN2 as well as DRP1 have been assessed in models of TBI. Due to the fact that both MFN1 and MFN2 are indispensable in mitochondrial fission, we may contextualise our results to MFN as a whole. Similar to the decrease in MFN we observed, Di Pietro et al., (2017) reported that in a model of severe TBI a decrease in MFN1 expression together with an increase in DRP1 expression was observed post-injury, whilst Martorell-Riera et al*.,* (2014) detected no significant changes in MFN1, a decrease in MFN2 and an increase in DRP1 in a model of increased excitotoxicity. Furthermore, in a model of ischaemia reperfusion, DRP1 expression increased whilst a decrease in MFN was observed (Kumari et al. 2012). These data suggest that upon neuronal injury, the equilibrium existing between fission and fusion events shifts more towards fission which results in increased mitochondrial dysfunction and ultimately cell death. It is generally accepted that upon injury, mitochondrial fission is induced (Fischer et al. 2016; Salman et al. 2021; Simmons et al. 2020; Q. Wu et al. 2018; S. Wu et al. 2011). We therefore propose that the change in DRP1 expression upon exposure to AF16 (Fig. 3.3) contributes towards its protective effects by maintaining mitochondrial function and ultimately decreasing cell death triggered by neuronal injury. Mitochondrial respiration analysis may be required in order to fully elucidate the impact of AF16 exposure on mitochondria.

The secondary injury associated with TBI results in an imbalance between the presence of oxidant and anti-oxidant agents, a phenomenon known as oxidative stress (Rodriguez-Rodriguez et al. 2014). Oxidative stress causes increased lipid peroxidation (LP), which results in the formation of a neurotoxic by-product known as 4-Hydroxynonenal (4-HNE), which further increases ROS production and oxidative protein damage (Hill et al. 2017). An increase in 4-HNE expression was observed post-injury (Fig. 7.5), which is in accordance with Mustafa et al*.,* (2010) and Rama Rao et al*.,* (2018), who also observed an increase in 4-HNE protein expression as early as 15 min post-injury. Of note, upon exposure to AF16, a tendency towards decreased 4-HNE expression was observed in comparison to control cells, especially at 36 h post-injury within the 35 kDa HNE adduct. A similar decrease in 4-HNE protein expression levels was observed by Mustafa et al., (2010) in a mouse model treated with a peroxyl radical scavenger. Therefore, this decrease in 4-HNE protein expression suggests a possible decrease in oxidative stress upon AF16 exposure.

Finally, we wished to assess how the signal and localisation of these mitochondrial proteins changed in proximity to the region of injury at each respective time point. Our results revealed that at 6 h post-injury MFN signal intensity (Fig. 7.2 C, D) was robustly lower than DRP derived signal intensity (Fig. 7.4) for both treatment groups; however, the increase in DRP signal intensity compared to MFN signal intensity over time in control cells was substantially higher than in AF16 treated cells (Fig. 7.2 and 7.4). To our knowledge, no prior studies have assessed the relationship between MFN and DRP signal intensity within the proximity of the wound boundary. AF16 may hence be able to preserve the equilibrium between mitochondrial fission and fusion, restoring mitochondrial function after neuronal injury, as early as 6 h post-injury. These findings are supported by the decrease in 4-HNE signal intensity observed in AF16-treated cells over time (Fig. 7.6 A–F). A decrease in 4-HNE within the wound area may be indicative of a decrease in oxidative stress and subsequent lipid peroxidation post-injury within cells treated with AF16. In studies done by Mustafa et al., (2010) and Singh et al*.,* (2013), a decrease in the levels of lipid peroxidation post-injury was observed together with the preservation of mitochondrial function and a decrease in the adverse effects caused by the secondary injury. Therefore, decreasing the levels of LP post-injury may provide protection post-injury.

## Conclusion

**Fig. 8:**
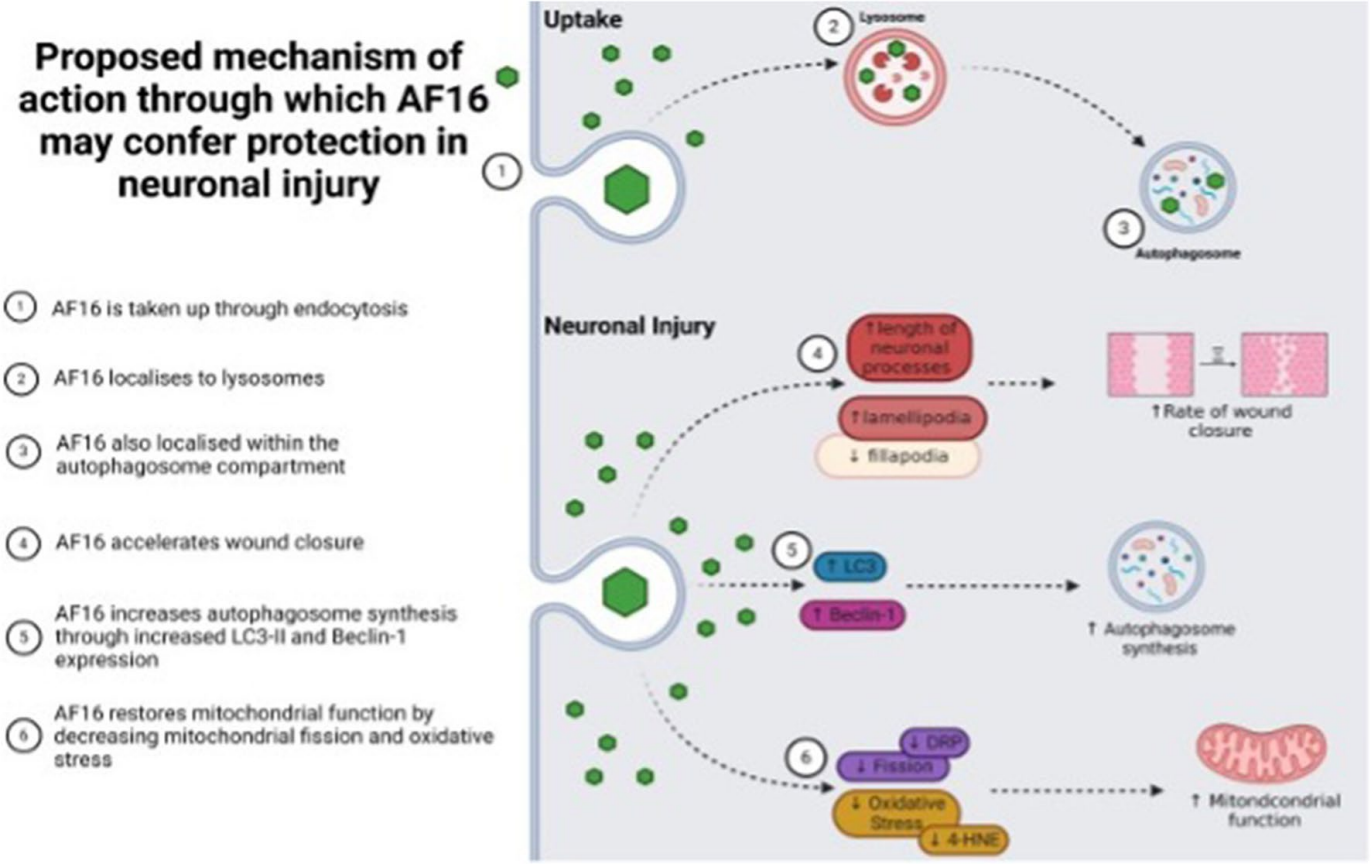
Key findings: AF16 is rapidly taken up intracellularly and localizes to the lysosomal compartment. AF16 exposure accelerates wound closure, enhances autophagosome synthesis, and decreases lipid oxidation

Traumatic brain injury (TBI) results in a delayed secondary injury response, causing the activation of pathological cascades ultimately resulting in cell death. Antisecretory factor (AF16) has shown promise in providing protection from at least some of these processes which would typically result in cell death; however, its mechanisms of action remained unclear. This study therefore aimed to characterise AF16 peptide uptake and to further investigate its effect on neuronal injury and the molecular mechanisms through which it may operate. Results obtained in this study showed that AF16 uptake is time dependent and AF16 localises to lysosomes and the autophagy compartment. AF16 exposure improves neuronal migration and morphology. Moreover, AF16 exposure increases autophagy synthesis and initiation, decreases oxidative stress, and maintains a favourable equilibrium between mitochondrial dynamics (Fig. 8). These findings suggest that AF16 may serve as a potential therapeutic agent for the treatment of TBI. However, additional studies using translational as well as in vivo model systems are urgently required, to clarify further the role of AF16 peptide in the neuronal injury response.

## Supplementary Information

Below is the link to the electronic supplementary material.

Supplementary file1 (PPTX 14862 KB)

## Data Availability

No datasets were generated or analysed during the current study.
