# Supplementary material for: Antisecretory Factor 16 (AF16): A Promising Avenue for the Treatment of Traumatic Brain Injury—An In Vitro Model Approach
Source: J Mol Neurosci. 2024 Nov 7;74(4):106. doi: 10.1007/s12031-024-02268-6 (PMC11541381; doi:10.1007/s12031-024-02268-6)
Supplement: Supplementary file 1 — Supplementary file1 (PPTX 14862 KB) [file 12031_2024_2268_MOESM1_ESM.pptx]

## Slide 1
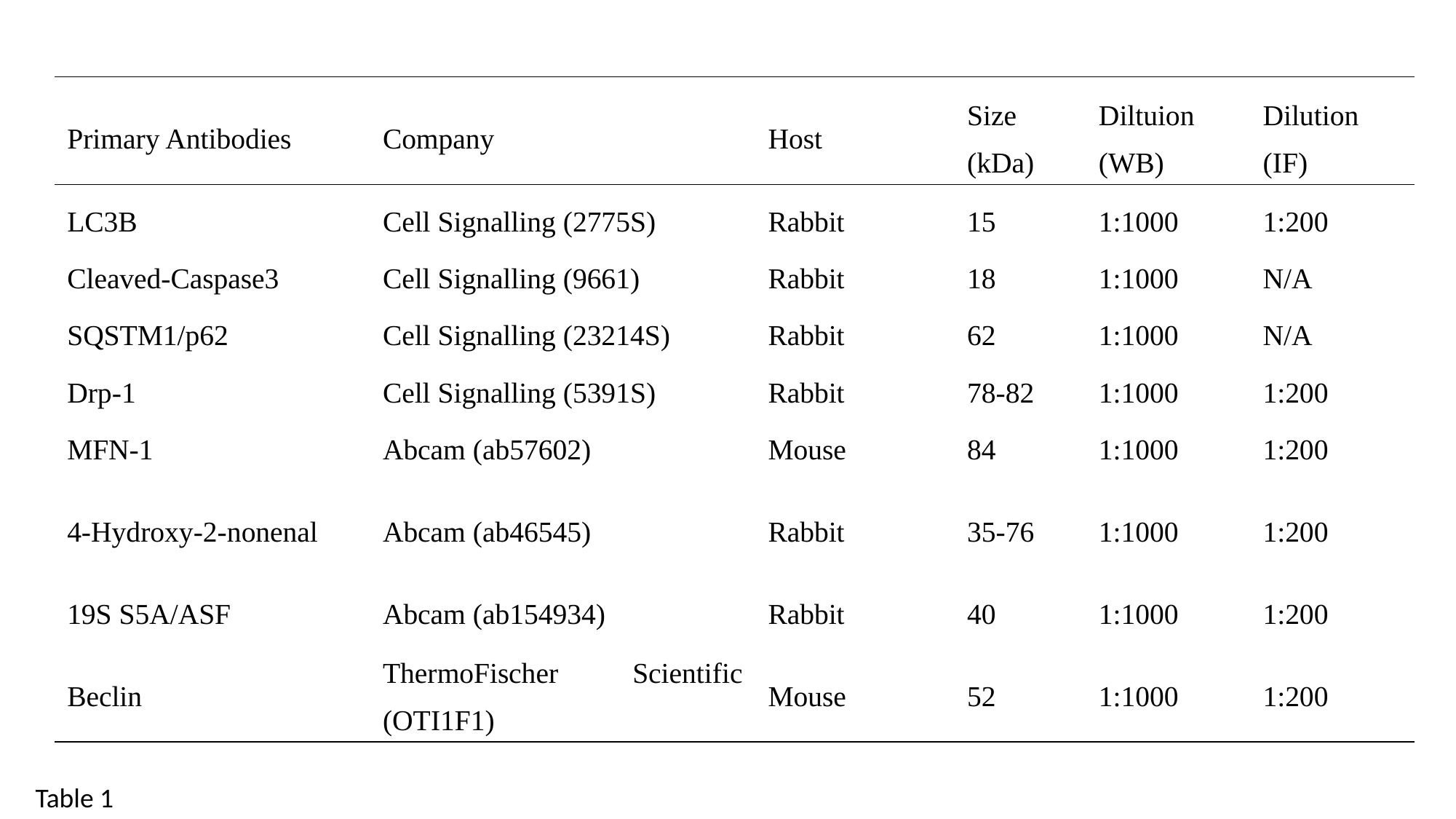

| Primary Antibodies | Company | Host | Size (kDa) | Diltuion (WB) | Dilution (IF) |
| --- | --- | --- | --- | --- | --- |
| LC3B | Cell Signalling (2775S) | Rabbit | 15 | 1:1000 | 1:200 |
| Cleaved-Caspase3 | Cell Signalling (9661) | Rabbit | 18 | 1:1000 | N/A |
| SQSTM1/p62 | Cell Signalling (23214S) | Rabbit | 62 | 1:1000 | N/A |
| Drp-1 | Cell Signalling (5391S) | Rabbit | 78-82 | 1:1000 | 1:200 |
| MFN-1 | Abcam (ab57602) | Mouse | 84 | 1:1000 | 1:200 |
| 4-Hydroxy-2-nonenal | Abcam (ab46545) | Rabbit | 35-76 | 1:1000 | 1:200 |
| 19S S5A/ASF | Abcam (ab154934) | Rabbit | 40 | 1:1000 | 1:200 |
| Beclin | ThermoFischer Scientific (OTI1F1) | Mouse | 52 | 1:1000 | 1:200 |
Table 1

## Slide 2
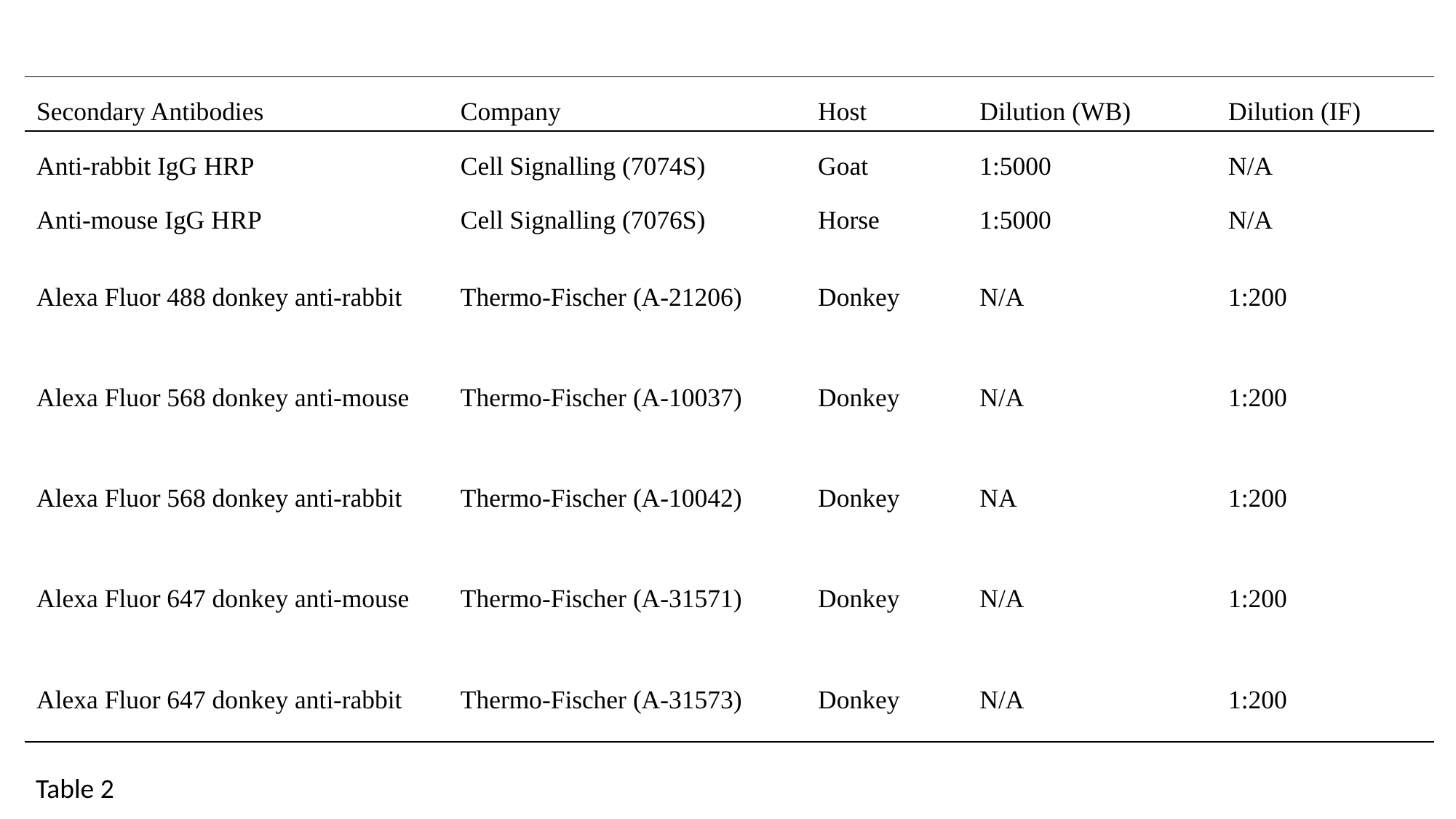

| Secondary Antibodies | Company | Host | Dilution (WB) | Dilution (IF) |
| --- | --- | --- | --- | --- |
| Anti-rabbit IgG HRP | Cell Signalling (7074S) | Goat | 1:5000 | N/A |
| Anti-mouse IgG HRP | Cell Signalling (7076S) | Horse | 1:5000 | N/A |
| Alexa Fluor 488 donkey anti-rabbit | Thermo-Fischer (A-21206) | Donkey | N/A | 1:200 |
| Alexa Fluor 568 donkey anti-mouse | Thermo-Fischer (A-10037) | Donkey | N/A | 1:200 |
| Alexa Fluor 568 donkey anti-rabbit | Thermo-Fischer (A-10042) | Donkey | NA | 1:200 |
| Alexa Fluor 647 donkey anti-mouse | Thermo-Fischer (A-31571) | Donkey | N/A | 1:200 |
| Alexa Fluor 647 donkey anti-rabbit | Thermo-Fischer (A-31573) | Donkey | N/A | 1:200 |
Table 2

## Slide 3
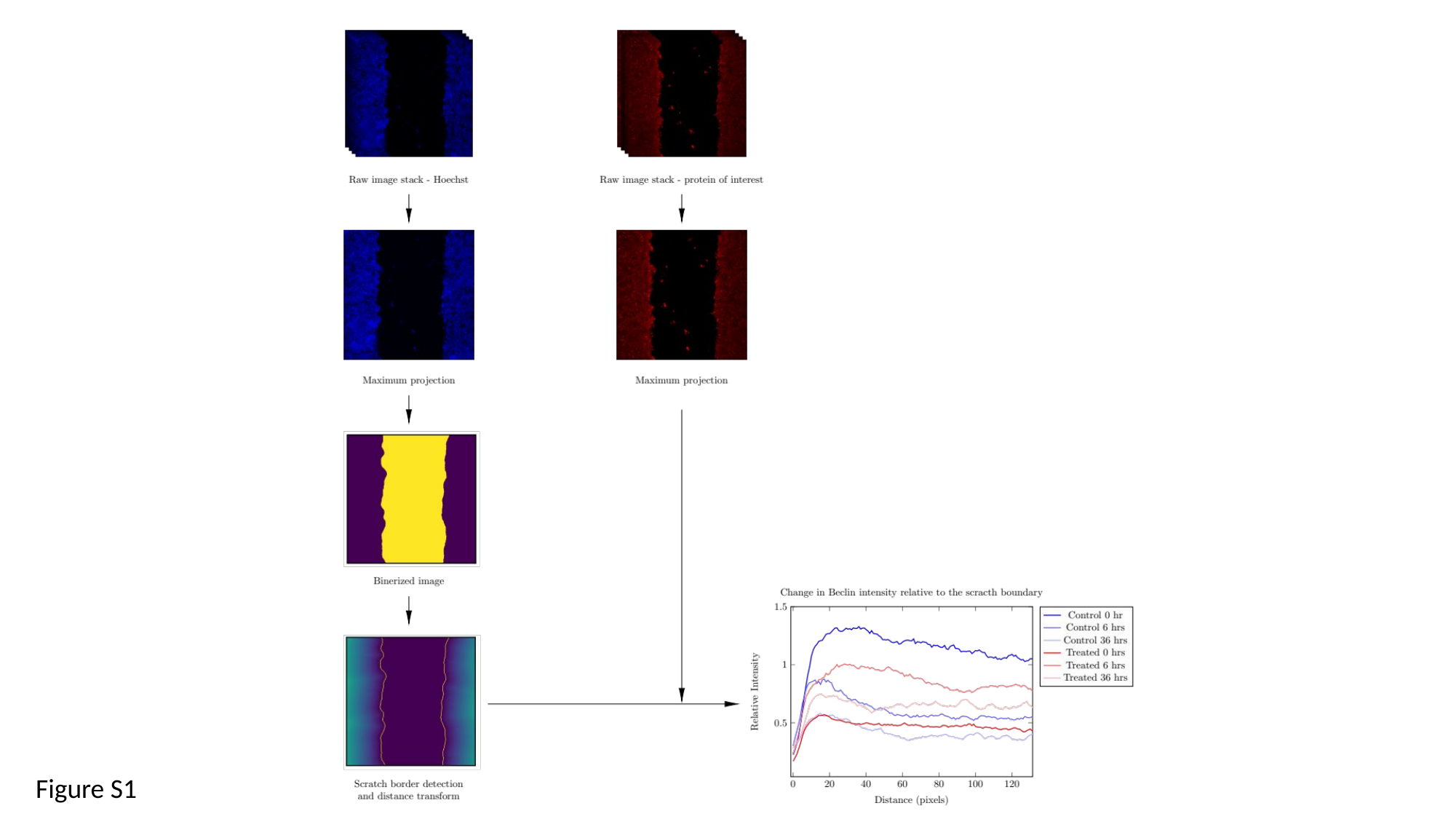

Figure S1

## Slide 4
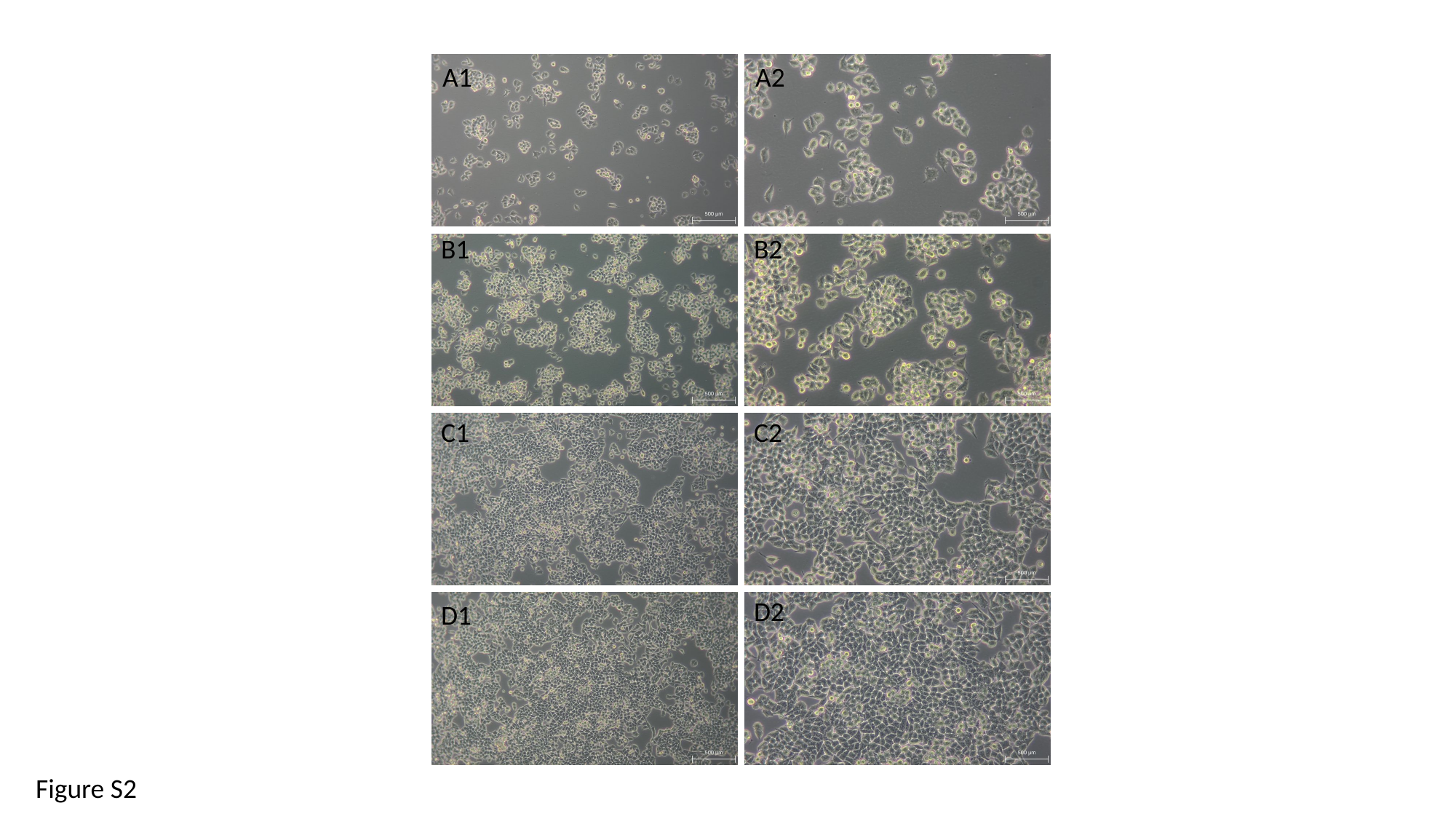

A1
A2
B1
B2
C1
C2
D2
D1
Figure S2

## Slide 5
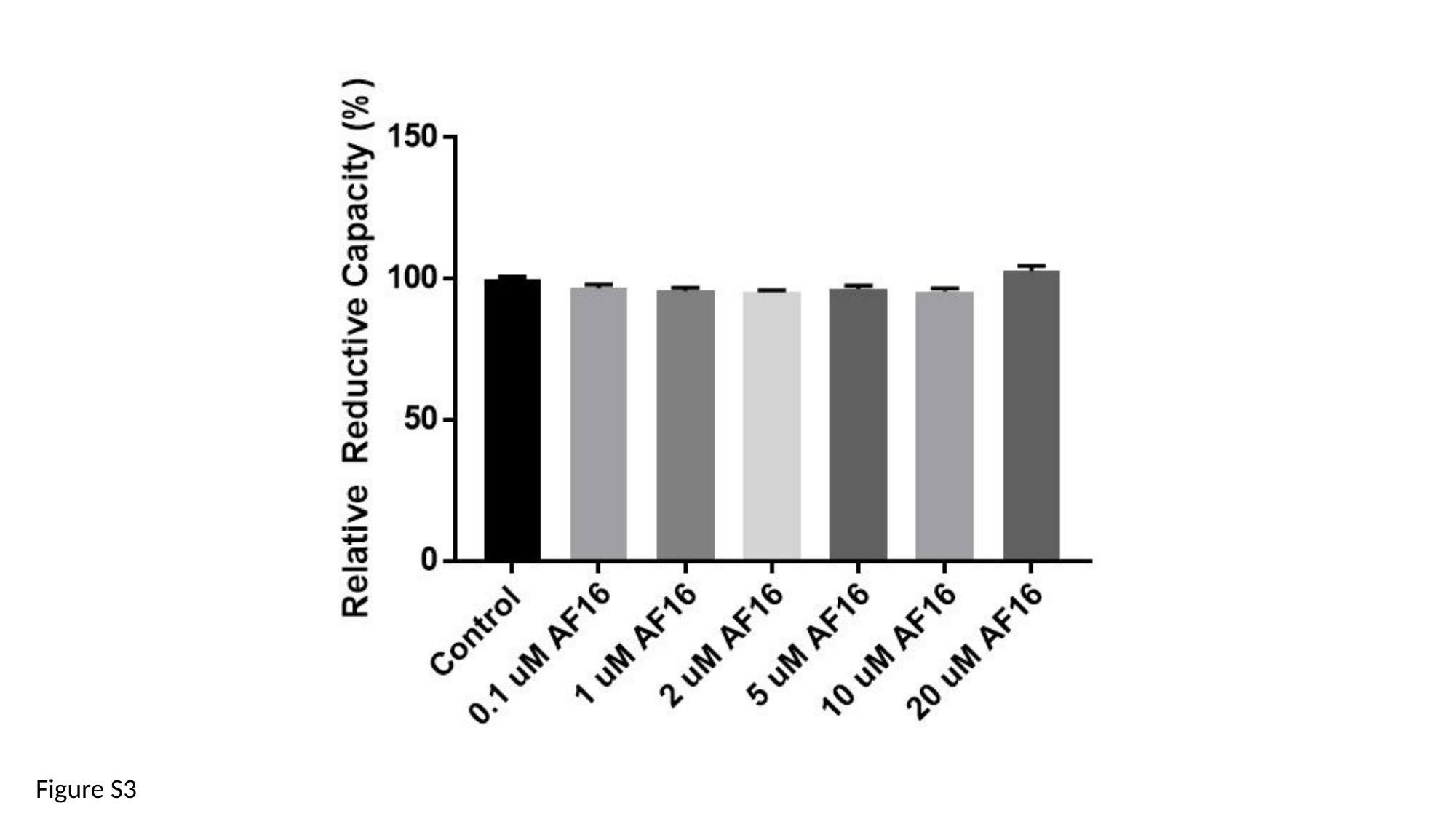

Figure S3

## Slide 6
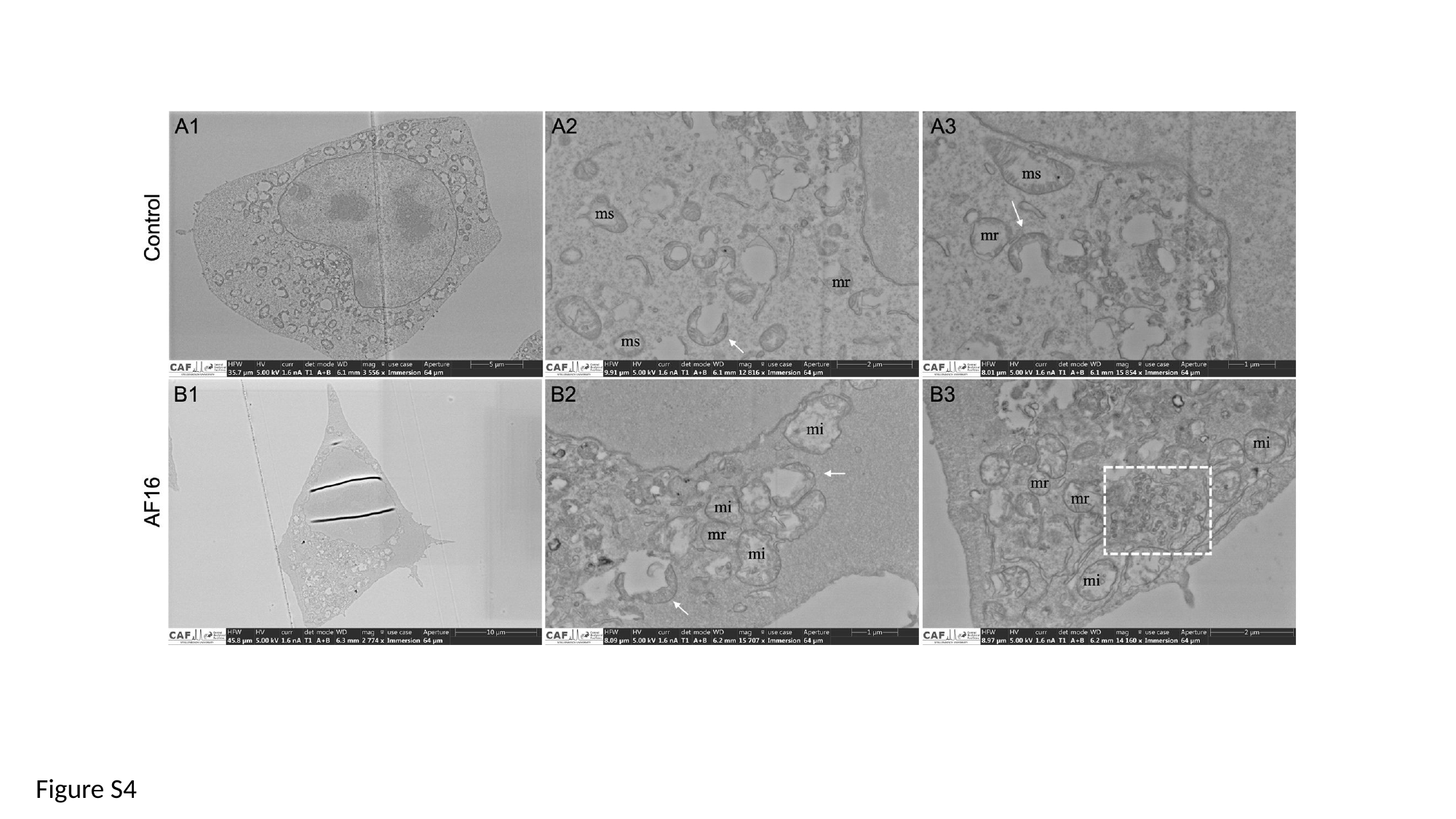

Figure S4

## Slide 7
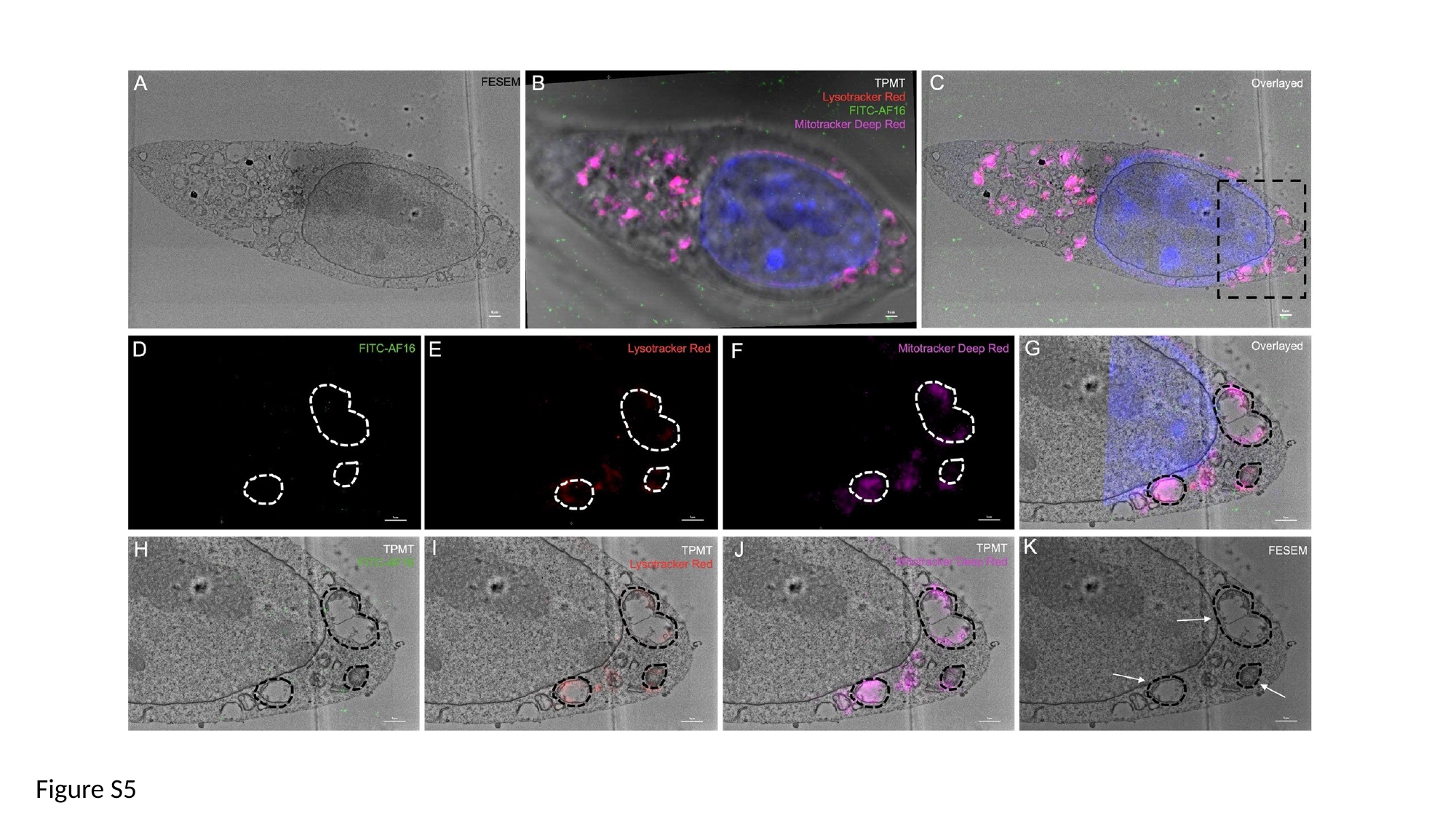

Figure S5

## Slide 8
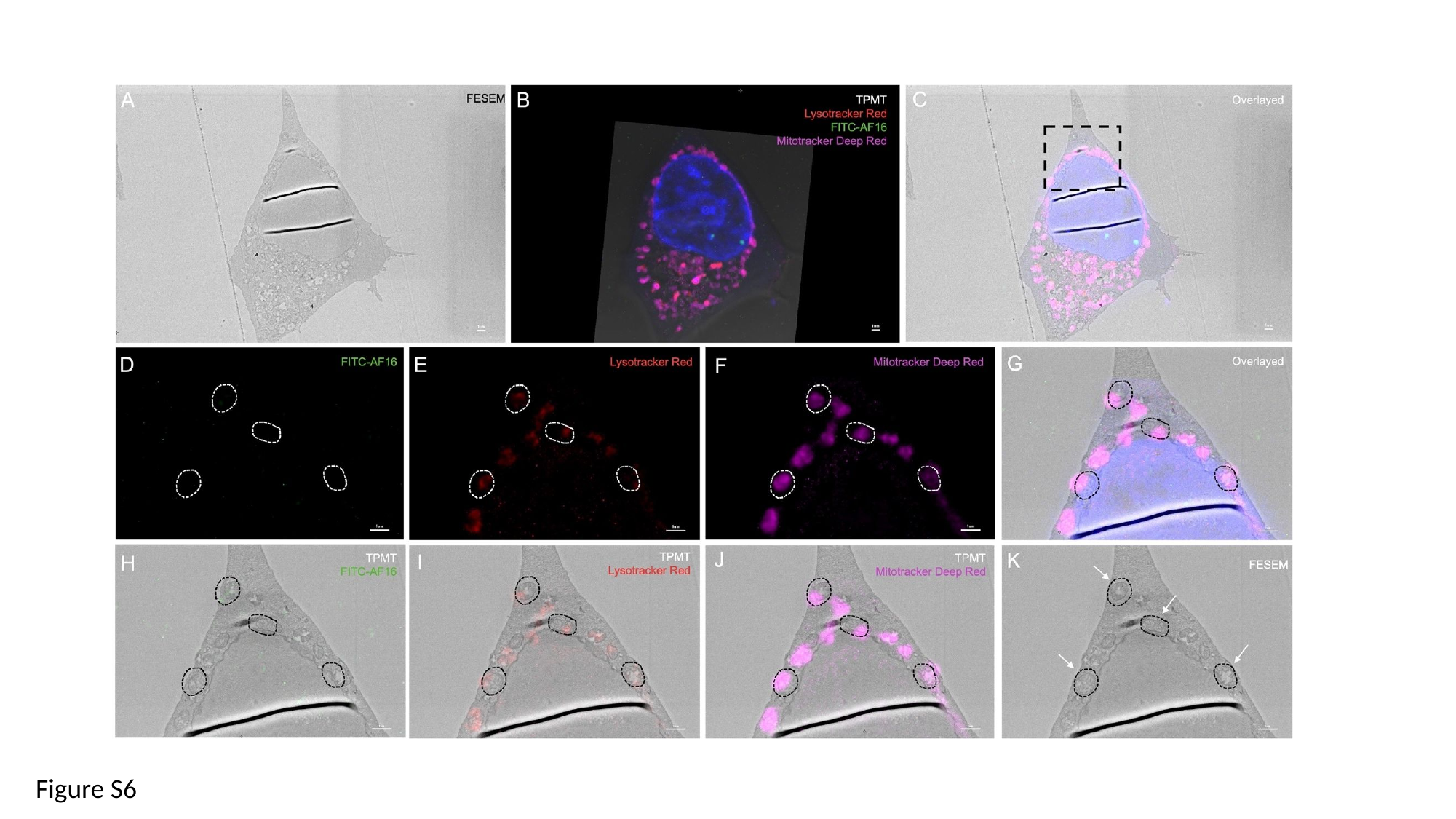

Figure S6
